# Systematic review of the effectiveness of menstrual health interventions in low- and middle-income countries in the East Asia and Pacific region

**DOI:** 10.1016/j.lansea.2023.100295

**Published:** 2023-10-19

**Authors:** Alexandra Head, Chelsea Huggett, Pisey Chea, Brooke Yamakoshi, Heather Suttor, Julie Hennegan

**Affiliations:** a Maternal, Child and Adolescent Health Program, Burnet Institute, Melbourne, VIC, Australia; b WaterAid Australia, Melbourne, VIC, Australia; c UNICEF East Asia and Pacific Regional Office, Bangkok, Thailand; d Melbourne School of Population and Global Health, University of Melbourne, Melbourne, Australia

**Keywords:** Menstrual health, Menstrual hygiene, Adolescent girls, Women's health, Dysmenorrhea, Systematic review, Health policy

## Abstract

**Background:**

In the context of rapidly expanding policy and practice, this systematic review collates and appraises evidence for the effectiveness of menstrual health interventions in the East Asia and Pacific region.

**Methods:**

Structured searches were undertaken in 7 databases and Google Scholar. Grey literature was identified through searching and survey of stakeholders. Quantitative evaluations were eligible. We audited the interventions and outcomes assessed in current evidence, undertook risk of bias assessment, and narrative synthesis of findings. The review protocol was registered prior to searching (PROSPERO: 343613).

**Findings:**

Eighteen studies were eligible; categorised according to the requirements for menstrual health they addressed. Information and education intervention studies (n = 11) found school-based programs improved menstrual knowledge test scores but did not evaluate impacts on broader outcomes. Evaluations of interventions providing materials, facilities, and services for menstruation (n = 4) focused on product acceptability. Studies exhibited a serious risk of bias without adequate controls, limitations in intervention allocation, adherence, and participant retention. Six studies of interventions to improve care for menstrual discomforts found decreased self-reported pain but had serious bias without placebo controls. Two interventions targeted the supportive social environment for menstruation.

**Interpretation:**

There is insufficient evidence for the effectiveness of menstrual health interventions in the East Asia and Pacific region. Future research must improve reporting, provide clear intervention theory of change, and improve measurement of core concepts. Evaluations of interventions that align with policy and practice are needed, facilitated by partnerships between researchers, government, and practitioners.

**Funding:**

UNICEF. Reckitt Global Hygiene Institute. 10.13039/501100000925NHMRC.

**Research in context**

**Evidence before this study**

Menstrual health has been recognised as essential for women's health and gender equality. Over the last decade, efforts to support menstrual health in the East Asia and Pacific region have advanced rapidly. However, there is a paucity of evidence to inform action. A 2019 systematic review of qualitative research on the menstrual experiences of women and adolescent girls in low-and-middle-income countries (LMICs) found only eight of 76 included studies from the East Asia and Pacific region. A past systematic review of interventions for menstrual health in LMICs, published in 2016 found no eligible evaluations in the region, nor did a 2016 review undertaken by UNICEF which further noted the absence of sufficient menstrual health research. The rapid advancement of menstrual health action means an updated review is warranted. Further, engagement with policy makers and practitioners has highlighted the primacy of region and country-specific findings in influencing policy and practice. This review accompanies a regional review of policy and programming, justifying our region-specific approach. Other developments in the field include the 2021 menstrual health definition that delineates the requirements for achieving menstrual health. Previous reviews differentiated only between ‘hardware’ (materials and facilities) and ‘software’ (education) interventions.

**Added value of this study**

This systematic review is the first to use the definition of menstrual health as a framework for collating and synthesising menstrual health interventions. The definition framework can be applied in future reviews. This systematic review is also the first to provide a review of evaluations in the East Asia and Pacific region. A region-specific review is in urgent need to inform the rapidly emerging developments in governmental and practitioner MH policy and programming. Our review was undertaken alongside an assessment of progress in policy and practice, enabling us to assess the evidence in the context of regional action and priorities. We found that interventions evaluated to date do not reflect current policy and practice. Further, our findings highlight that most evaluations in the region have focused on proximal outcomes related to menstrual product acceptability or knowledge but have yet to include measures of menstrual health or broader health and education outcomes. Researchers and policy makers can use the review to understand current evidence progress, the limited evidence for interventions, and to address current limitations in future research.

**Implications of all the available evidence**

There is insufficient evidence for menstrual health interventions in the East Asia and Pacific region. Partnership between policy makers, practitioners and researchers is needed to rigorously evaluate interventions aligned with current policy and programming focus. Future evaluations must outline clearer theories of change and include menstrual health and broader outcomes. In absence of evidence, interventions in the region should be implemented with strong monitoring and evaluation frameworks.

## Introduction

Menstrual health (MH) impacts half of the global population for up to 40 years of their lives. Yet, MH challenges have been under researched and under acknowledged. MH is gaining greater attention and is increasingly recognised as essential to achieve gender equality and the sustainable development goals.^1^ Despite this, millions of women, adolescent girls, and people who menstruate struggle to achieve it.^2^, ^3^, ^4^, ^5^ Effective interventions are needed across contexts; to improve MH and associated outcomes. This systematic review aimed to appraise the current evidence for the effectiveness of MH interventions in the East Asia and Pacific region, to inform policy and practice, and identify gaps.

MH is defined as “a state of complete physical, mental, and social well-being and not merely the absence of disease or infirmity, in relation to the menstrual cycle.”^6^ The definition establishes five requirements for MH: i) access to information and education about the menstrual cycle across the life-course; ii) access to materials, facilities and services to care for the body such that preferences, hygiene, comfort, privacy and safety are supported, iii) access to timely diagnosis, treatment and care for menstrual cycle related discomfort and disorders, iv) access to a supportive social environment free from stigma and psychological distress; and v) non-discrimination and freedom to participate in all spheres of life throughout the menstrual cycle.^6^ Interventions to improve MH are likely to do so by targeting one or more of these requirements.

This review used the definition as a framework to categorise the types of evaluated interventions and synthesise the evidence for each requirement. Our approach evolves the intervention typologies used in past systematic reviews of MH or menstrual hygiene management (MHM) interventions. These typologies focused on ‘hardware’ (the provision of physical resources such as menstrual materials or sanitation infrastructure) and ‘software’ (the provision of education or social programs).^3^^,^^5^^,^^7^ Further, our review provides an update for the East Asia and Pacific region. Searches for past systematic reviews were undertaken in 2012^7^ and 2015.^5^ Neither identified any evaluations of interventions in the region. Similarly, a comprehensive 2016 UNICEF^8^ review of progress and action for menstrual hygiene management programming across schools, out-of-school youth, community and humanitarian contexts in East Asia and the Pacific identified no evaluations across the 17 included countries.

Synthesising the evidence for the effectiveness of MH interventions in the East Asia Pacific region is timely, due to increased policy attention and development of programmes.^9^ Our systematic review serves as a companion to a regional review of progress in policy and practice, allowing us to contextualise research progress alongside current policy and service delivery efforts. The regional review included a desk review of policy, survey, and interviews with stakeholders, synthesised in a regional report.^9^ The advisory group for the regional review provided oversight for this systematic review, and a stakeholder survey supported grey literature searching.

## Methods

The review protocol was registered prior to searching (PROSPERO registration number: 343613)^10^ and is reported in compliance with the Preferred Reporting Items for Systematic Review and Meta-Analyses (PRISMA) statement (see Supplementary Materials 1).^11^

### Eligibility criteria

Studies published in any language were included, however searches were undertaken in English. We searched for studies from 2015 onwards, as two previous systematic reviews, one published in 2016, had not identified any studies in this region prior to this date. Thus, we expect identified studies to represent the full body of evidence to date.

#### Study design

All quantitative study designs were eligible. This included: randomised controlled trials (RCTs), Cluster randomised controlled trials (cRCTs), controlled before-after studies (CBA), non-randomised trials (studies including a non-randomised, investigator-assigned control group with endline-only outcome assessment), case–control studies comparing exposed and unexposed participants, and uncontrolled pre-post or endline-only evaluations.

#### Population

Studies were eligible if they reported on outcomes for menstruating women, girls or other people who menstruate from 19 low and middle-income countries in UNICEF's East Asia and Pacific region:

1 Fiji
2 Federated States of Micronesia
3 Kiribati
4 Papua New Guinea
5 Samoa
6 Solomon Islands
7 Vanuatu
8 Cambodia
9 Indonesia
10 Lao People's Democratic Republic
11 Mongolia
12 Myanmar
13 Philippines
14 Timor-Leste
15 Viet Nam
16 People's Republic of China
17 Democratic People's Republic of Korea (DPRK)
18 Malaysia
19 Thailand

#### Interventions

Studies testing interventions addressing the requirements for MH outlined in the definition were eligible.^6^ Table 1 describes how each requirement was operationalised and the corresponding eligible interventions.

**Table 1:** Eligible interventions according to the requirements for MH.

Information and education	Interventions that provided education or access to information about the menstrual cycle, menstrual care or puberty were eligible. We considered interventions providing broader puberty or sexual and reproductive health education eligible if they reported including components related to the menstrual cycle and menstruation.
Materials, facilities, and services	Interventions designed to improve access to menstrual materials, either disposable or reusable, for example through free product provision were eligible. Interventions that improved water, sanitation, and hygiene (WASH) facilities or menstruation-friendly facilities such as through provision of water supply, soap, or private toilets. We considered interventions providing broader WASH interventions eligible if they reported objectives related to improving facilities for menstrual hygiene/self-care.
Care for discomforts and disorders	Eligible interventions were those designed to improve access to or availability of health care for menstruation. This included: efforts to train health care workers in menstrual topics or improve care quality, improve knowledge relevant to seeking treatment, or training to enable care for discomforts such as in medication selection or self-care strategies such as yoga, exercise, breathing or stretching techniques. Clinical trials comparing pharmaceutical or homeopathic remedies in their effectiveness at reducing menstrual pain were not eligible. Additionally, we excluded studies of acupuncture or health care provider-administered treatments focused on assessing only the effectiveness of pain reduction. Initial database searches identified studies that focused on pain relief that could be delivered at the community level such as stretching or yoga. These were categorised self-care interventions that equip the participant with the knowledge or practice to self-administer care for menstrual related discomfort or pain and were considered eligible.
Supportive social environment	We considered eligible interventions which aimed to improve social support or dismantle stigma or harmful norms surrounding menstruation. We anticipated such interventions would be informed by social and behavioural theories and include components beyond education alone. Interventions to improve social support could also include education delivered to support sources such as teachers or parents, rather than intended beneficiaries themselves.
Non-discrimination and participation	The regional review^9^ of MH included this requirement with attention to legal frameworks to dismantle discrimination or facilitate participation. Such frameworks are unlikely to be evaluated, and we hypothesised that improved participation may be facilitated by interventions addressing the above requirements. Evaluations of such policy interventions would be considered eligible, however preliminary searching identified no such evaluations. We did not include this requirement in results.

#### Control/comparator

Any control group, including waitlist, treatment as usual or comparison to another intervention were eligible.

#### Outcomes

We did not exclude studies based on outcomes. A-priori we anticipated including outcomes which aligned with the integrated model of menstrual experience,^10^ including those assessing improvements to contributors to menstrual experiences (e.g., knowledge, behavioural expectations, social support), menstrual experience (e.g., feelings of shame and distress, menstrual practices, perceptions of practices and environments), and broader consequences of poor MH including physical health (reproductive tract infection and irritation), mental health, education, employment, and social participation.

### Stakeholder engagement

An advisory group (n = 9) of technical experts and stakeholders from the East Asia and Pacific region provided feedback across three online meetings on (i) the review questions, methods, and stakeholder mapping; (ii) preliminary country-level findings and analytic approach; and (iii) draft regional synthesis findings and dissemination strategies.

### Search methods

#### Database searches

The search strategy (see Box 1) was developed prioritising sensitivity, with assistance from a librarian and drawing on past reviews and pilot searches.^2^^,^^5^^,^^7^^,^^12^ Searches were undertaken in June 2022 in seven electronic databases: ASSIA (Applied Social Science Index and Abstracts), Cochrane Central Register of Controlled Trials (CENTRAL), CINAHL, EMBASE, MEDLINE, ProQuest Dissertations and Theses, and PsycINFO. Google Scholar was searched by combining menstrual health search terms together with the name of one included country, exporting the first ten results. This process was repeated for each country, as well as the two regions, totalling 21 Google Scholar searches and 210 results. This varied from the pre-registered protocol as Google Scholar did not allow long search strings, thus each country was searched individually.

Box 1 **Illustrative database search strategy.**

**Table:** 

#	Search term
1	Menstruation/
2	(menstru∗ or menses or catamenia or menarche or dysmenor∗ or endometrios?s or amenor∗ea or menor∗agi∗ or oligomenor∗ or premenstrual syndrome).mp.
3	(menst∗ adj3 (period or cycle or disorder∗ or pain∗)).mp.
4	(period? adj1 (pain∗ or disorder∗ or irregular∗ or infrequent or abnormal)).mp.
5	(heavy period? or light period∗ or period discomfort∗).mp.
6	1 or 2 or 3 or 4 or 5
7	Location specification. See appendices for complete list.
15	7 or 8 or 9 or 10 or 11 or 12 or 13 or 14
16	6 and 15
17	controlled clinical trial/or randomized controlled trial/
18	cohort studies/or controlled before-after studies/
19	(randomi?ed controlled trial or controlled trial or control trial or controlled study or control group? or experimental study or non-randomi?ed trial or pilot trial or randomi?e? or trial or randomly).mp.
20	(cohort or controlled before-after or “before-after study” or pre-post evaluation∗ or endline evaluation∗ or endline or “difference-in-differences” or pretest or posttest).mp.
21	(Evaluation OR comparison).mp.
22	(Program∗ OR policy OR intervention).mp.
23	17 or 18 or 19 or 20 or 21 or 22
24	16 and 23
25	limit 24 to yr = "2015–Current"

Full search strategies reported in Supplementary Materials 2.

#### Additional searching

In August 2022, keywords (including “menstrual∗” “menstruation” “menstrual health” “menstrual hygiene” AND “intervention” “evaluation”) were searched in grey literature repositories including the WHO Library, the Sustainable Sanitation Alliance (SuSanA) knowledge Hub, the Menstrual Health Hub, and Menstrual Hygiene Day resources library. We searched websites of organisations working on MH, such as member organisations of the Pacific Menstrual Health Network and Global Menstrual Collective, along with those identified through stakeholder mapping undertaken with the advisory group. Surveys and interviews with stakeholders, as part of the regional review^9^ further supplemented electronic searching.

The reference lists of eligible studies and recent UNICEF reports^8^^,^^13^ were searched. Citation tracking using Google Scholar was undertaken for eligible studies and UNICEF reports including the 2016 regional review.^8^^,^^13^

### Data collection and extraction

Two authors (AH, JH) independently screened titles, abstracts, and full texts using the JBI Summari platform.^14^ Discrepancies were resolved through discussion. A data extraction form was developed and piloted to capture study characteristics, intervention details, and outcome measurement. One author (AH) extracted study data which was reviewed by a second author (JH).

### Risk of bias assessment

The risk of bias for RCTs was assessed using Risk of Bias 2 (RoB2)^15^ or RoB2 for clustered trials.^15^ For non-randomized studies, the Risk of Bias in Non-randomized Studies of Interventions (ROBINS-I) tool was used. RCTs were rated as low, some concerns or high risk of bias across five domains: randomization process, deviations from intended interventions, missing outcome data, measurement of outcomes, and selection of the reported result^15^; and additional sixth domain (recruitment of participants into clusters) for clustered designs.^16^ For non-randomised studies, studies were rated as low, moderate, serious, or critical risk of bias based on seven domains: confounding, participant selection, intervention classification, deviation from interventions, missing data, measurement of outcomes, and selective reporting.^15^ For consistency across the two tools, classifications were relabelled as ‘low, moderate (previously some concerns), serious (previously high risk) or critical.’ Due to the poor quality of evidence and high heterogeneity we did not undertake an assessment of certainty in the body of evidence.

### Synthesis methods

First, given the broad scope of the review, information was tabulated to provide an overview of study locations, study types, participants, interventions, outcomes and measures, and to highlight similarities and differences.^17^ Studies were grouped according to the MH requirement(s), i) access to information and education, ii) access to materials, facilities and services, iii) access to timely diagnosis, treatment and care, iv) access to a supportive social environment. Within each grouping, we undertook a narrative synthesis to describe (1) the types of interventions evaluated (what was being done), (2) the coverage and comparability of outcomes assessed (what outcomes were being measured), and (3) study findings in the context of bias (what was found and was it credible). Heterogeneity in intervention and outcomes, and inadequate statistical reporting (e.g., failure to report standard deviations), meant we were unable to undertake meta-analysis (see Supplementary Materials 6). The narrative synthesis prioritised studies with lower risk of bias in reporting study effect estimates.^17^

### Ethics approval

The Alfred Health Research Ethics Committee approved the survey of regional stakeholders (project number: 360/22). All participants gave informed consent before completing the survey. This review uses only published works.

### Role of funding source

This study was primarily funded by UNICEF with support from WASH thematic funding. UNICEF co-author (B. Yamakoshi) and stakeholders acknowledged as part of the study advisory group, contributed to the review design and interpretation of findings. The corresponding author had full access to all the data in the study and had final responsibility for the decision to submit for publication. The findings, interpretations, and views expressed in this publication do not necessarily reflect the views of UNICEF.

## Results

**Fig. 1:**
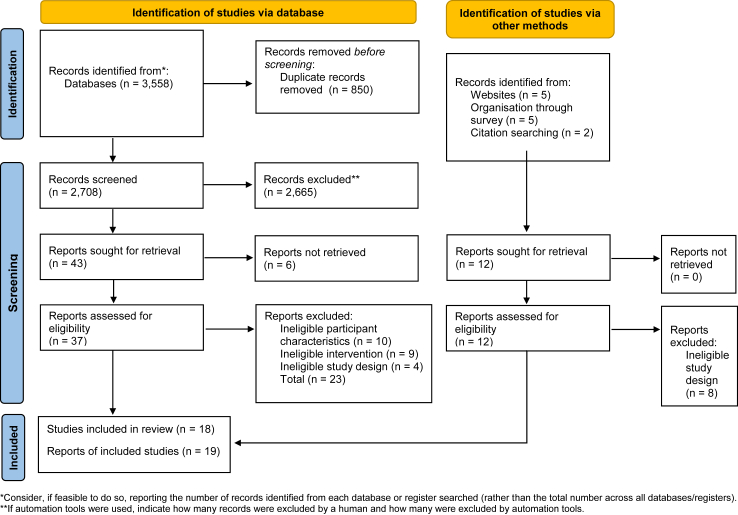
**PRISMA**^11^**searching and screening flow diagram**.

Fig. 1 presents the results of searching and screening.

### Included studies

Eight of the 19 countries included in the review were represented in 18 eligible studies. Indonesia had the highest number of studies (n = 7), followed by China (n = 2), Vietnam (n = 2), Malaysia (n = 2), Vanuatu (n = 2) and one study in Laos, Mongolia, and Thailand. Most studies focused on adolescent girls (n = 14), with 12 specifically on school students. Five studies of school students included boys. Six studies included various adult women: university students (n = 2), migrant workers (n = 1), health care workers (n = 1), athletes (n = 1) and communities in a natural disaster setting (n = 1).

**Fig. 2:**
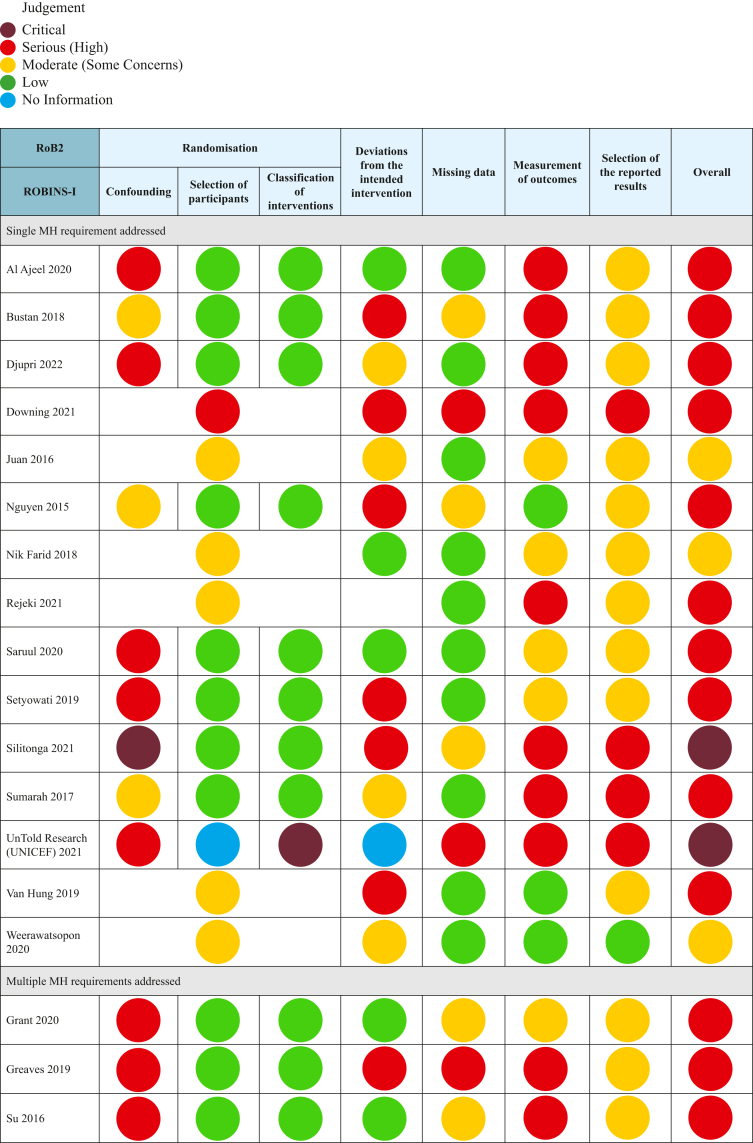
**Risk of Bias summary**.

Study characteristics are presented in Table 2, and risk of bias in Fig. 2 with full assessment provided in Supplementary Materials 3. Four studies included interventions addressing multiple requirements for MH and were included in syntheses across multiple categories. Fig. 2 presents a summary of the risk of bias assessment.

**Table 2:** Characteristics of included studies.

Study ID	Requirement/s for MH addressed	Country	Year of study	Study design	Participants	Study aim, intervention and control conditions	Time to follow up	Outcomes, and methods of assessment
**Single MH requirement addressed**								
Al Ajeel 2020^18^	Care for discomforts and disorders.	Malaysia.	2017–2018	CBA	**Total:** 480 (Intervention: 268, Control: 212) Adolescent girls (students) with Primary Dysmenorrhea Age 13–18.	**Test effectiveness of Primary Dysmenorrhea (PD) Education on knowledge and self-care.** **Intervention:** Participants received a 30-min health education session covering menstruation, PD and PD self-care methods. Lecture, debate, exchange of experiences and PD information pamphlet were used to deliver the program. **Control.** No intervention. Booklet provided at end of study.	2 months post-intervention.	**PD Knowledge**: Score on 20-item Knowledge of Primary Dysmenorrhea (KoPD) scale.^19^^,^^20^ **PD Self-care**: Score on 40 item Adolescent Dysmenorrhea Self-Care scale (ADSCS) (6-subscales: 4 items in searching for knowledge, 6 items for expressing emotions, 4 items in seeking assistance, 7 items in control over external factors, 14 in resource utilisation, 5 items in self-control).^21^
Bustan 2018^22^	Care for discomforts and disorders.	Indonesia.	Not reported	CBA	**Total:** 96 (Intervention: 48, Control: 48) Female nursing students. Experienced PD in past 6-months. Age 18–19. Rural Setting.	**Test effectiveness of abdominal stretching for decreasing PD pain.** **Intervention:** Participants received instructions on how to conduct abdominal stretching. Participants undertook 10–15 min of abdominal stretching twice weekly for three weeks. **Control:** No intervention.	After the 3-week intervention period.	**Pain**: Self-rated on Likert scale (0–5).
Djupri 2022^23^	Care for discomforts and disorders.	Indonesia.	Not reported	CBA	**Total:** 58 (Intervention: 29, Control: 29) Female students. Age 16–17. Urban Vocational School.	**Test effectiveness of pelvic rocking and Buteyko breathing exercises on reducing PD pain.** **Intervention:** Intervention content not reported. Participants received 30-min sessions across two days. **Control:** Not reported. Assumed no intervention.	Immediate.	**Pain**: Self-rated on Likert scale (0–10).
Downing 2021^24^	Materials, facilities, and services.	Vanuatu.	2019	RCT	**Total:** 192 (136 follow up). Women and girls in disaster effected settings. Age 15–45. One rural and one urban setting.	**Compare experience and preferences for menstrual products for inclusion in MHM kits in disaster settings.** **Intervention/comparison 1:** One packet Maxfree disposable pads were provided. Participants were given 2-months to trial pads. Products were provided along with a kit (containing clothesline and pegs, underwear x 2, soap, and detergent), with verbal information on use from research team. **Intervention/comparison 2**: One packet of Modess disposable pads were provided. Participants were given 2-months to trial pads. Products were provided along with a kit, and verbal education. **Intervention/comparison 3**: One packet Mumma's Laef reusable pads were provided. Participants were given 2-months to trial pads. Products were provided along with a kit, verbal education and manufacturers written instructions. **Intervention/comparison 4**: One packet AFRIpads reusable pads were provided. Participants were given 2-months to trial pads. Products were provided along with a kit, verbal education and manufacturers written instructions.	2 months post-intervention.	**Acceptability of sanitary product**: Assessed using an investigator developed 6-item (fit, comfort, leakage, smell, changing, likelihood of recommending) questionnaire. The study was mixed methods, qualitative feedback on product use experience collected.
Juan 2016^25^	Care for discomforts and disorders.	China.	2014–2015	cRCT	**Total:** 391 (Intervention 195, Control: 196) Female university students with PD. Age 18–20.	**Test effectiveness of self-management education to improve PD symptoms and quality of life (QoL).** **Intervention:** Participants received self-management education covering knowledge about PD, safe medication, non-pharmacological treatment (massage, pressure points, acupuncture) menstrual care, exercise, diet, social activities, psychological regulation, emotional, and interpersonal management. The intervention was delivered over 2-months, through lectures, group and individual counselling sessions, and a series of workshops. Participants then carried out self-management for 6-months. **Control:** No intervention.	Followed up for 6 months (observation period).	**PD symptoms**: Score on Dysmenorrhea Symptom questionnaire based on Dysmenorrhea Symptom Criteria in the Guidelines for Clinical Research on New Chinese medicines.^26^ **Pain**: Self-rated on the Visual Analogue Scale (VAS) (0–10) **QoL**: Score on 8-item Chinese version^27^ of the SF-36 scale.
Nguyen 2015^28^	Information and education.	Vietnam.	Not reported	Non-randomised trial	**Tota**l: 928 (Intervention: 459, Control: 469) Adolescent school students from 1 urban and 1 rural school.	**Test effectiveness of an Sexual and Reproductive Health (SRH) education session to improve puberty and SRH knowledge.** **Intervention:** 45-min reproductive health education lecture, with opportunities for questions and discussion. Presumed to be delivered by investigators. Menstruation included as part of SRH education. No further details on education content were reported. Outcomes indicate topics included puberty, reproductive systems, fertility, and possibly the menstrual cycle. **Control:** No intervention.	2 weeks post-intervention.	**SRH Knowledge**: Correct responses to investigator-developed knowledge quiz items (no. items not reported). Knowledge test items related to the menstrual cycle included: o Knowledge about the signs of puberty o Knowledge of fertile window
Nik Farid 2018^29^	Information and education.	Malaysia.	2013–2015	RCT	**Total:** 209 (Intervention: 101, Control: 108) Adolescent school students. Aged 12. Urban setting.	**Compare effectiveness of SRH Education delivery methods for SRH knowledge and attitudes.** **Intervention:** Module of 17 topics, including menstruation, female reproductive systems, and physical development during puberty delivered online. After an introduction, participants had 1.5-h to navigate the website containing videos, informational graphs, and articles. **Comparison:** Module of 17 topics, including menstruation, female reproductive systems, and physical development during puberty. Modules were delivered by a public health specialist as a 3-h in-person educational session (conventional method), using flyers and group discussions.	Immediate.	**SRH Knowledge**: Score on quiz (no. items not reported) developed by investigators based on WHO illustrative questions for asking young people about SRH.
Rejeki 2021^30^	Care for discomforts and disorders.	Indonesia.	Not reported	RCT	**Total:** 130 (Intervention: 65, Control: 65) Female adolescent students. Aged 15–17. Urban and rural settings.	**Test effectiveness of abdominal stretching for reducing PD pain.** **Intervention:** The intervention is not reported. Based on the reported information it is likely that participants undertook abdominal stretching for 10–15 min. Number of sessions not reported. **Control:** Not reported. Assumed no intervention.	Not reported.	**Pain**: Self-rated on Likert scale (0–10).
Saruul 2022^31^	Information and education.	Mongolia.	2020	Pre-post	**Total:** 86 (71 post-menarchal). Adolescent girls in 8th Grade from two schools. Rural setting.	**Test effectiveness of health education and menstrual hygiene training for improving menstrual knowledge.** **Intervention:** Researchers delivered the health education and menstrual hygiene training using a PowerPoint presentation. Training was reviewed and approved by Health Education teachers. Duration not reported. **No control group**.	Immediate.	**MHH Knowledge**: Score on investigator-developed 10-item knowledge quiz. Investigators also explored endline experiences of menarche, emotional state during menstruation, and menstrual hygiene practices, but did not compare these outcomes to baseline or a control.
Setyowati 2019^32^	Information and education.	Indonesia.	2018	CBA	**Total:** 174 (Intervention: 87, control: 87). Premenarchal adolescent girls from Madrasah–Islamic school. Aged 9–12. Acehnese. Rural setting.	**Test effectiveness of a Menarche Preparations Reproductive Health Education booklet on participants' knowledge, emotional response, and attitude towards menarche.** **Intervention:** Researchers provided a booklet containing information on the reproductive organs, physical changes during adolescence, problems during menstruation, management strategies, and menstrual hygiene. **Control:** No intervention (waitlist). Booklet provided after study completion.	Not reported.	**Menstruation & Puberty Knowledge**: Score on 14-item quiz used in past research.^33^^,^^34^ **Emotional response**: Score on a 13-item Emotional Responses Scale used in past research.^35^^,^^36^ **Attitude towards menstruation**: Assessed using the 13-item Adolescent Menstrual Attitude Questionnaire.^33^^,^^37^
Silitonga 2021^38^	Information and education.	Indonesia.	Not reported (Ethics approval obtained 2019)	CBA	**Total:** 24 (Group A: 8, Group B: 8, Group C: 8) Female migrant workers, pre-departure. Age reported as: Adults (63% < 30, 29% 30–35, 8% > 35). Urban setting.	**Compare effectiveness of SRH education including varied illustrative “case” content on SRH knowledge and attitudes.** **Intervention 1:** Four 120-min sessions, twice a week for two weeks. SRH education on topics including reproductive anatomy, pregnancy and abortion, sexuality and sexual abuse, and sexually transmitted infections. MH topics included within the SRH education were female reproductive organs, fertile window, and menstruation. Module included 1–3 cases (assumed by review authors to mean “scenarios”). **Intervention 2:** The same four 120-min sessions as Intervention 1. Module included 5+ cases. **Comparison/Control:** The same four 120-min sessions as Intervention 1. No cases.	Not reported.	**SRH Knowledge and Attitudes**: Investigator developed test of 70% knowledge and 30% attitude questions (no. of items not reported). No further information is reported regarding the measures used. The study was mixed methods, qualitative feedback on education sessions collected.
Sumarah 2017^39^	Information and education.	Indonesia.	2016	CBA	**Total:** 80 (Intervention: 40, Control: 40) Adolescent girls from two schools. Majority Urban.	**Test effectiveness of vaginal hygiene education on vaginal hygiene practice, attitudes and behaviours.** **Intervention:** Participants were given 6-months to access and independently explore an online self-learning module on maintaining general vaginal hygiene (including menstrual practices such as changing panty liners, choosing menstrual pads, and changing menstrual pads). No further information on education content was reported. **Control:** No intervention.	6-months post intervention.	Evaluation included broader Vaginal Hygiene outcomes; MHH-related outcomes included: **Vaginal Hygiene Behaviour**: Correct responses to investigator developed questionnaire (no. questions not reported) capturing: • Changing panty liners • Choosing menstrual pads • Changing menstrual pads **Vaginal Hygiene Attitudes**: Correct responses to investigator developed questionnaire (no. questions not reported) capturing: • Changing panty liners • Choosing menstrual pads • Changing menstrual pads
Van Hung 2019^40^	Information and education.	Vietnam.	Not reported	RCT	**Total**: 400 (Intervention: 200, Control: 200). Male and female ethnic minority students. Age not reported. Rural and mountainous setting.	**Test effectiveness of seminars on puberty and SRH knowledge for improving reproductive health knowledge.** **Intervention:** Three 45-min seminars, run weekly. Participants received a 15-min lecture using videos, pictures and scenarios followed by 15-min small group discussions, and 15-min feedback sessions. Education package designed by Hanoi National University of Education. MH topics included knowledge of the reproductive system, puberty stages, and stages of the menstrual cycle. **Control:** No intervention.	6-months post-intervention.	**SRH Knowledge**: Correct responses to university developed and approved knowledge test items (no. not reported). Knowledge test items related to the menstrual cycle included: o Timing of menarche o Knowledge of fertile window
Weerawatsopon 2020^41^	Materials, facilities, and services.	Thailand.	2019–2020	Randomised Crossover Trial	**Total**: 98 (Group A: 49, Group B: 49) Female health care personnel. Age 18–50 (x̄: 32). Urban Hospital setting.	**Compare satisfaction and acceptability of menstrual cups and sanitary pads through a self-delivered intervention.** **Intervention:** Menstrual cups and sanitary pads were provided to all participants to trial over 6-months. Participants selected one of two sized menstrual cups. An instruction manual and an instructional video were provided to all participants. Group A trialled sanitary pads for cycles 1st—3rd and menstrual cups for 4th—6th cycles. Group B trialled the products in the reverse order.	At 3-months and 6-months (post intervention).	**Product satisfaction**: Assessed using an investigator developed 10-item (insertion, removal, leakage, odour prevention, cleaning, on land, in water, daily activities, sleep comfort, overall satisfaction, frequency of changing) questionnaire. Removal for cleaning and adverse reactions reported individually. **Acceptability of Menstrual Cup**: Assessed at 6 months only.
**Multiple MH requirements addressed**								
Grant 2020^42^	Information and education. Materials, facilities, and care. Supportive social environment.	Laos.	2017–2019	Pre-post	10 Schools **Total:** 292 (Midterm: 291, Endline: 286). Adolescent school students. Aged 10–19. Rural setting.	**Test effectiveness of Menstrual Health and Hygiene (MHH) education as part of a wider WASH program for improving menstrual knowledge, attitudes, social support, and social participation** **Intervention:** Provision of WASH services (i.e.: separate male and female toilets, waste pits, safe drinking water, soap and handwashing stations) and a life-cycle healthy living education program. MH was included as one component of the lifecycle education program. Red Cross Teacher Volunteers and Red Cross Youth are trained in the effective delivery of programming and youth clubs are formed. Participants received a 1-day MHH education workshop including reproductive age, puberty, adolescent reproduction, hormones, physical change in the body. An additional 4-h training on how to sew reusable pads was provided. **No control group.**	Varied (Intervention ran in 10 schools between 2017 and 2019. Endline conducted end of 2019).	Evaluation included broader WASH outcomes; MHH-related outcomes included: **School absenteeism due to menstruation:** Self-reported through a single question. **MHH Knowledge and Attitudes:** Score on 7-item quiz including knowledge and attitude questions. **Supportive environment:** Self-reported through investigator developed survey with single questions capturing: o Talking to a teacher before menarche o Talking to someone before menarche
Greaves 2019^43^	Information and education. Materials, facilities, and services.	Vanuatu.	2019	Pre-post	**Total:** 82 (25 follow-up) Female athletes. Age 13–59 (x̄ = 22) (89% 13–29).	**Test uptake and satisfaction with menstrual products and effects of education and products on sport participation.** **Intervention:** Participants received an in-person MHM education session on basic anatomy, the female reproductive system, detailed menstrual cycle education, pain management, period-tracking, myths and taboos, and instruction on use and care for various menstrual products. The duration was not reported. The participants chose from either 2 x pairs of reusable menstrual underwear or 1 pair of reusable menstrual underwear and 1 menstrual cup to use over a period of 4 months.	After the 4-month trial period.	**Missing sport due to period:** Self-reported through a single question. Qualitative feedback on education sessions and product use experience also collected.
Su 2016^44^	Information and education. Care for discomforts and disorders.	China.	Not reported	CBA	**Total:** 128 (116 final sample) (Intervention: 66, Control: 62). Post-menarchal adolescent girls from two schools. Aged 12–15. Urban setting.	**Test effectiveness of culturally and developmentally tailored MH education sessions for improving menstrual knowledge, attitudes, confidence, and health behaviours.** **Intervention:** Investigator delivered grade-level appropriate, interactive sessions guided by Kolb's Experimental Learning Theory including lectures, experience sharing, group discussion, and visual aids through five weekly 45-min education sessions. Topics included menstruation, hygiene practice, nutrition, pain management, and differentiation between normal conditions and problems that might require medical assistance. Participants were able to provide anonymous feedback after each session and the teaching plan was modified accordingly. **Control:** Wait-list control.	Immediate.	**Menstrual Knowledge and ability to pursue and use information on menstruation:** Score on a 13-item Menstrual Knowledge Questionnaire used in past research.^45^^,^^46^ **Menstrual Attitude:** Score on 5-item Chinese version of 7-item Menstrual Attitudes Questionnaire (MAQ).^47^, ^48^, ^49^ **Confidence for Menstrual Health Care:** Score of 5-item Menstrual Health Care Behaviour Questionnaire (MHCBQ).^50^ **Pain Self-Care behaviour:** Score on 18-item Dysmenorrhea Related Self-care Behaviour Questionnaire (DRSCBQ).^46^^,^^51^
UnTold Research (UNICEF) 2021^52^	Information and education. Supportive social environment.	Indonesia.	2019–2020	CBA	25 schools: (Intervention: 20 Control: 5). Student sample: (Baseline: 911, endline 762). Urban setting.	**Test effectiveness of a multi-component MHM program including written resources, peer-leader training, Oky period tracker and educational app, teacher sensitization and community engagement activity on puberty and menstrual knowledge, menstrual hygiene practice, and menstrual product access.** **Intervention:** 1 MHM storybook provided to schools and made accessible to students. 2 MHM training was provided for: (a) Adolescent Health Cadres (student leaders), and (b) students. Education included awareness of menstrual hygiene and knowledge transfer along with life skills education. (c) Health care workers and (d) teachers received education sessions online, including MHM information and teaching strategies. 3 Trained Adolescent Health Cadres delivered menstrual hygiene sessions in 40 schools. Sessions included story book reading, group discussion, and introduction of the Oky period-tracker app. Optional activities included videos, developing banners or other arts based or participatory activities. 4 Competition held by UNICEF for a writing, photo or video submission related to MH. **Control: Not reported/unclear**.	Immediate.	Evaluation included broader health outcomes; MHH-related outcomes included: **SRH Knowledge**: Correct responses to investigator developed questionnaire (no. questions not reported) capturing: • Puberty signs **Menstrual Knowledge and Perceptions**: Correct responses to investigator developed questionnaire (no. questions not reported). **MHM**: Correct responses to investigator developed questionnaire (no. questions not reported) Mixed methods study, qualitative data also collected through observational surveys, interviews and focus groups. **Comfortable talking about puberty with someone.**

CBA: Controlled before-after study; RCT: individually randomized controlled trial; cRCT: cluster randomized controlled trial; x̄: mean.

### Information and education

#### Interventions

11 studies evaluated information and education interventions. Five were exclusively MH education interventions,^31^^,^^32^^,^^39^^,^^44^^,^^52^ five included MH as part of broader educational programming^28^^,^^29^^,^^38^^,^^40^^,^^42^ and one included a MH education session to support the delivery of menstrual product provision.^43^ Across studies, education session duration varied from a single 45-min session to 6-months unlimited access to an online learning module. Two studies provided educational booklets (Setyowati 2019,^32^ Untold 2021^52^).

The exclusively MH interventions ranged in focus and content. In Indonesia, Setyowati 2019^32^ utilized a booklet to support menarche preparedness, Sumarah 2017^39^ tested an online self-learning hygiene module and Untold 2021^52^ provided online MH student leader training accompanied by a storybook. Education sessions were iteratively tailored based on student feedback in China (Su 2016^44^); and PowerPoint supported MH training in Mongolia (Saruul 2022^31^).

The five studies that incorporated MH as part of a wider program, SRH (n = 4) or WASH (n = 1) focused on puberty, reproductive anatomy, and pregnancy, and did not describe the MH-specific content included in their intervention. Like Sumarah 2017,^39^ Nik Farid 2018^29^ tested an online learning module, while others tested variations of in-person workshops. One intervention which included the provision of reusable menstrual products, focused its education content to train on product use, period tracking and dismantling myths and taboos (Greaves 2019).^43^

#### Outcomes

Most evaluations (n = 9/11) measured knowledge as the single or primary outcome. Knowledge measure content and quality varied widely (see Table 2 and Supplementary Materials 6). Some studies used a continuous quiz score, whilst others selected a cut-off value with limited justification. Two studies integrated questions capturing attitudes within their knowledge measure (Silitonga 2021^38^; Grant 2020^42^). Evaluations of MH-specific education included more questions capturing knowledge related to self-care strategies, whereas SRH education studies most often included a smaller range of questions relevant to MH, such as knowledge of the signs of puberty and the fertile window.

Three studies included an independent ‘menstrual attitudes’ outcome.^32^^,^^39^^,^^44^ Su 2016^44^ used the Menstrual Attitudes Questionnaire^47^ capturing perceptions of menstruation as debilitating or bothersome, whereas Sumarah 2017^39^ tested attitudes towards practices and behaviours, such as changing pads. Two studies assessed participant perceptions of social support or comfort discussing menstruation with others, and two assessed menstrual-related absenteeism from school or sport.^42^^,^^43^

#### Risk of bias

Of the 11 studies, two were assessed as critical,^38^^,^^52^ eight as serious^28^^,^^31^^,^^32^^,^^39^^,^^40^^,^^42^, ^43^, ^44^ and one as having a moderate risk of bias.^29^ Generally, all studies lacked sufficient reporting across multiple domains, such as method of and allocation concealment, measurement of outcomes or selection of the reported result. Only Nik Farid 2018^29^ mitigated some concerns by reporting randomisation uncompromised by contamination due to clustering. Three studies without control groups, Grant 2020,^42^ Greaves 2019^43^ and Saruul 2022,^31^ were rated as serious risk of bias. The nature of education interventions results in an inability to blind participants and was a risk across studies. However, the impact of blinding varies across outcomes. Self-reported attitudes and behaviours, particularly those reported immediately following the education session, are more likely to be impacted by social desirability bias than knowledge tests, and as such received differing assessments of associated risk of bias.

#### Effects of interventions

All studies measuring knowledge as an outcome reported improvements following the intervention, most often measured immediately after the education session. Three studies, all MH-focused education, reported sufficient data on the change in a continuous knowledge score, Saruul 2022^31^ and Su 2016^44^ both reported an approximate 30 percentage point improvement in the intervention group, compared to pre-intervention or the control group respectively. The third, Untold Research (UNICEF) 2021^52^ reported a 7 percentage point improvement, though was compromised by substantial participant drop out. Three education interventions with no-intervention controls (Setyowati 2019,^32^ Van Hung 2019,^40^ Nguyen 2015^28^), all with a broader focus of SRH content, reported an improvement in correct responses or ‘good’ knowledge of 13–32 percentage points in favour of the intervention. Interventions testing teaching modalities demonstrated slight differences in favour of the intervention, including online compared to in-person and using case-studies compared to without. Individual study findings are summarised in Supplementary Materials 6. The only studies testing longer term retention (Van Hung 2019,^40^ 6-months and Nguyen 2016,^28^ 2-weeks), had increased likelihood of contamination due to intervention and control groups attending the same school.

Three studies evaluating changes in menstrual attitudes reported improvements post-intervention across varying scales. Su 2016^44^ reported improvements across subscales of attitudes describing menstruation as debilitating [pre: 2.81 (SD 1.07) post: 3.00 (SD 1.09)] in the intervention group, while both intervention [pre 2.70 (SD 1.10) post: 2.99 (SD 1.08)] and control [pre: 2.52 (SD 1.20) post: 2.73 (SD 1.15)] groups improved in finding menstruation bothersome. Setyowati 2019^32^ did not report pre-intervention control scores but reported the intervention group scoring at 54.1 percentage points higher than the control on ‘positive attitudes’ towards menstruation. Adequate controls could have aided to mitigate self-report biases. Together, findings indicated that providing education improved knowledge and attitudes, but the effect sizes and impacts on broader outcomes were either not evaluated or exhibited a serious risk of bias.

### Materials, facilities, or services

#### Interventions

Four studies^24^^,^^41^, ^42^, ^43^ evaluated interventions seeking to improve access to materials, facilities, or services for MH. Three studies focused on comparing the provision of different menstrual products to adult women, to a no-treatment control: Weerawatsopon 2020^41^ compared the provision of a menstrual cup or disposable pads for health care workers (HCW) in Thailand, Downing 2021^24^ compared the provision of disposable or reusables pads for women impacted by disasters as part of a hygiene kit in Vanuatu, and Greaves 2019^43^ compared providing a menstrual cup or reusable menstrual underwear to athletes aged 13–59 in Vanuatu alongside a menstrual education session. Grant 2020^42^ was the only intervention providing access to facilities through separate male and female toilets, waste-pits, and hand-washing stations in school settings, in addition to a 4-h training on sewing reusable menstrual pads for adolescent girls in Laos.

#### Outcomes

Both Downing 2021^24^ and Weerawatsopon 2020^41^ assessed product satisfaction and acceptability through Likert-scales. A lack of standardized measures for product acceptability (e.g., fit, comfort, leakage, or cleaning) impacted comparability. Broader impacts on MH or health and education were not assessed, with the exception of Weerawatsopon 2020^41^ in which participants self-reported genital irritation. Greaves 2019 and Grant 2020^42^ measured effects on absenteeism from sport and from school respectively, both using a single self-report question.

#### Risk of bias

Weerawatsopon 2020^41^ was appraised with some concerns for risk of bias, while Downing 2021,^24^ Grant 2020,^42^ Greaves 2019^43^ were all rated serious (high). Weerawatsopon 2020^41^ reported participant randomisation, blinding analysts, and low loss-to-follow-up. Both Downing 2021^24^ and Weerawatsopon 2020^41^ mitigated some concerns of self-report bias in the acceptability appraisals through comparison of two menstrual products rather than a no-treatment control in the case of Greaves 2019.^43^ All studies were limited by inadequate reporting on participant allocation, allocation concealment, and adherence to interventions. Downing 2021^24^ and Greaves 2019^43^ reported significant loss-to-follow-up, with Greaves 2019^43^ only reporting endline data for 30% of the sample. Lack of transparency in the reported participant numbers and statistical analyses used prompts caution in the interpretation of these studies.

#### Effects of interventions

Weerawatsopon 2020^41^ and Downing 2021^24^ reported high levels of product acceptability (>80%) for all products. Weerawatsopon 2020^41^ reported that HCWs in Thailand had statistically significant greater overall satisfaction with menstrual cups than disposable pads, with participants rating menstrual cups a median four, and sanitary pads three on a 5-point Likert scale. Fewer participants using menstrual cups (8.2%) reported genital symptoms (irritation, dermatitis) than those using pads (36.1%). Downing 2021^24^ tested two brands of disposable menstrual pads and two brands of reusable menstrual pads, finding a slight preference for one disposable brand (90% vs 80%) and one reusable brand (97% vs 86%) respectively. Greaves 2019^43^ measured participation in sport after providing an education session and either a menstrual cup or reusable menstrual underwear and found that the 30% of retained participants reported less absenteeism. Grant 2020^42^ reported high school attendance at baseline and no change to endline following the provision of a multi-component intervention including improvements to WASH facilities.

### Care for discomforts and disorders

#### Interventions

Six studies^18^^,^^22^^,^^23^^,^^25^^,^^30^^,^^44^ tested care for discomforts and disorders interventions, providing self-care exercises or education in Indonesia, China, and Malaysia. We found no studies evaluating interventions to improve accessibility of diagnosis or medical treatment or targeting the health care system. Two studies tested the effectiveness of interventions providing information on PD and self-care and management strategies: Juan 2016^25^ for university students with PD in China and Al Ajeel 2020^18^ for adolescents with PD in Malaysia. Su 2016^44^ tested the effectiveness of MH education sessions in China including pain management and identifying conditions that might require medical assistance. Bustan 2018,^22^ Djupri 2022^23^ and Rejeki 2021^30^ assessed primary dysmenorrhea (PD) pain levels after providing self-care strategies including, abdominal stretching^22^^,^^30^ or pelvic rocking and breathing exercises^23^ to female university or school students in Indonesia.

#### Outcomes

Four^22^^,^^23^^,^^25^^,^^30^ of the six studies assessed pain as the primary outcome, self-reported by participants on a Likert-scale. Education-focused interventions measured additional outcomes, including PD symptoms using clinical guidelines, QoL (Juan 2016)^25^ and enacting PD self-care practices (Al Ajeel 2020),^18^ Su 2016^44^ assessed confidence in self-care behaviours, in addition to MH knowledge, and the ability to source and use MH information.

#### Risk of bias

Juan 2016^25^ was appraised as moderate risk of bias as an cRCT. While the study was randomised, reporting of randomisation, allocation concealment and adherence were incomplete. The remaining five interventions were rated as a serious risk of bias.^18^^,^^22^^,^^23^^,^^30^^,^^44^ These five studies lacked information on the intervention dose and/or adherence. Self-reported pain outcomes and comparison to a no-treatment control rather than a placebo such as a sham intervention or placebo pill compromises the conclusions that could be drawn from the studies.

#### Effects of interventions

Juan 2016^25^ found that providing education resulted in reduced self-reported pain (standardised mean difference d = −0.62, 95% CI −0.82,−0.41), PD symptoms, and improved QoL indicating the potential of education and self-management interventions for improving MH across a more diverse set of outcomes (Supplementary Materials 6 reports individual study findings). Al Ajeel 2020^18^ and Su 2016^44^ also reported positive effects of education on PD knowledge (with a 15 percentage point improvement beyond the control), and self-reported self-care behaviours. Three studies^22^^,^^23^^,^^30^ assessing pain reduction through stretching, breathing, or pelvic rocking reported significant reductions in pain ranging from 1.5 to 2.11-point improvement over the control group, on numeric rating scales from 0 or 1 to 10. Studies did not report standard deviations, precluding meta-analysis.

### Supportive social environment

#### Interventions

Two studies (Grant 2020^42^ and Untold 2021^52^) included activities aiming to improve the social environment for girls. Both interventions included training of teachers and youth leaders, Grant 2020^42^ provided ‘peer-to-peer’ behaviour change modules for girls reaching menarche, alongside improvements to school WASH facilities. Untold 2021^52^ provided online training for teachers and health officers to informally pass on to students, while trained students implemented training in schools with a ‘peer-to-peer’ approach. The interventions sought to improve student comfort in discussing menstruation with a teacher or peers.

#### Outcomes

Grant 2020^42^ tested the effects of the MH activities on female students’ willingness to talk to a teacher or another person before reaching menarche. While, Untold 2021^52^ measured students comfort discussing menstruation with support people (including: sister, female friends, female teacher, health officer).

#### Risk of bias

Grant 2020^42^ was appraised as exhibiting a serious risk of bias across multiple domains, including outcome measurement. The study does not utilise a control group and measures a subjective outcome through self-report. Untold 2021^52^ was rated as critical risk of bias, as no study design or allocation method is reported, the intervention and control were not adequately described, and participant numbers are inconsistently reported.

#### Effects of interventions

Grant 2020^42^ found participants reported greater willingness to discuss menstruation with a teacher (0% baseline to 57% endline) or someone else (36% baseline to 49% endline) following the intervention. Untold 2021^52^ found girls exposed to intervention programming reported significantly higher agreement (58%, compared to a baseline of 53%) that they have a person they can trust to ask questions about their body, compared to girls attending control schools who showed no significant increase in agreement (51%, compared to a baseline of 53%). Intervention group increased self-reported comfort discussing menstruation with the following individuals, beyond the change reported in the control group: sister: 0%, mother: −8%, female friends: 3%, female teachers: 6%.

## Discussion

Through this systematic review we aimed to collate and synthesise current evidence for the effectiveness of interventions aiming to improve MH in the East Asia and Pacific region. We identified 18 quantitative evaluations of MH interventions published since 2015. Identified studies represent an stark increase in research on the topic; with no eligible studies identified in the region in any prior reviews.^5^^,^^7^^,^^8^ While this increased attention is promising, we found insufficient evidence of the effectiveness of MH interventions in meeting the requirements for MH or providing broader health or education outcomes. Research to date has been small-scale and exhibited a serious risk of bias. Most included studies evaluated the effects of interventions to address deficits in menstrual knowledge, followed by a small collection of studies investigating self-care strategies for menstrual pain, and a minority testing interventions addressing materials for menstruation, with a few interventions addressing multiple requirements. We used the requirements for MH outlined in the definition^6^ as a framework to synthesise the interventions. Our framework can provide a model for future reviews. Our systematic review was undertaken alongside a review of progress in policy and service delivery in the East Asia and Pacific region.^9^ Through our discussion we contextualise findings from the review considering policy and programming in the region; assessing alignment between policy and practice and the synthesised evidence base to enhance recommendations for research and practice.

### Information and education interventions

Menstrual knowledge is hypothesised to improve girls’ MH and reduce negative psychosocial consequences such as distress.^5^^,^^53^, ^54^, ^55^ No included studies assessed broader outcomes although they have been included in trials of MH interventions in other regions, such as a menstrual health trial in Uganda which included mental health and quality of life outcomes^55^^,^^56^ In the present review, interventions tested to improve MH knowledge were small-scale training initiatives delivered outside of formal education systems to school-aged females, and to a lesser extent, female university students. This differs from progress in the region,^9^ where our regional review of practice identified progress in policy objectives for MH education taught through school curriculums. We did not identify any evaluations of government curriculum or large-scale education programmes. Due to limited reporting on included education content, it was not possible to assess the extent of alignment between interventions evaluated and government curriculum or Comprehensive Sexuality Education guidelines.^9^^,^^57^ Future research should develop tools to report and appraise the comprehensiveness of MH education, for different age groups, for improved intervention reporting and comparison. This would enable stronger assessments of external validity. Moreover, future evaluations should consider the quality of content and delivery in the provision of education.

Recent review and audit of measures in MH research globally^58^ highlighted the absence of definitions for core MH concepts, such as MH knowledge, attitudes, and behaviours as barriers to measurement and study comparability. Similarly, our review found that the conceptualisation of menstrual knowledge in evaluations was unclear and varied, with questionnaires largely based on the educational content rather than against standards or domains of menstrual knowledge. Future research to define core menstrual knowledge and education content, paired with tools for assessing content coverage, would enable stronger evaluations.

### Materials, facilities, and services interventions

Review of progress in policy and practice in East Asia and Pacific highlighted that governments in the region are prioritising WASH in schools’ policies and guidelines to support MH. While 13 of 14 countries included in the regional review have WASH in Schools policies or guidelines that make provisions for MH, our systematic review of evaluations found only one study testing the effectiveness of these facilities for MH, which had serious risk of bias.

Few studies evaluated the effectiveness of material or facility provision for MH, and three of the four identified studies focused on the acceptability of menstrual products. In policy and practice throughout East Asia and Pacific, non-governmental organisations and governments are pursuing free or subsidised menstrual product provision. This review found no robust evidence for the effectiveness of this approach. Studies found products provided were acceptable to users. Such investigation represents an important first step, aligned with global discourse^59^, ^60^, ^61^ highlighting the importance of product quality and informed choice.^60^ However, high-quality evaluations are urgently needed to understand the effectiveness of product provision models on improving access to menstrual products, MH, and broader health and education outcomes to inform policy and practice. Trials in other geographic regions (Sub-Saharan Africa, South Asia) have trialled product provision including but not limited to school attendance, wellbeing, and reproductive health including urogenital infection, with mixed results to date.^5^^,^^7^^,^^55^ All interventions that tested product provision included reusable menstrual products, reflective of the growing attention to reusables and environmental sustainability.^9^^,^^62^

### Care for discomfort and disorders interventions

Care for discomfort and disorders has been more recently recognised as a core requirement for MH. Review of policy and practice in East Asia and Pacific found limited government policy or initiatives in this area. Our systematic review did not find any interventions that evaluated improved access to diagnosis, treatment, and provider-administered care. Eligible studies were more akin to clinical trials of pain relief effectiveness. Exclusively testing pain fails to elucidate how self-care education and strategies impact broader MH outcomes including school, university or work attendance, academic achievement, participation, or psychosocial outcomes. The included PD education studies (Juan 2016,^25^ Su 2016)^44^ tested additional outcomes, including likelihood to seek help when experiencing an issue or differentiation between normal conditions and conditions that might require medical assistance. Although there were methodological limitations, this provides a positive starting point for further attention. This systematic review, and the review of policy and practice suggests investment in developing interventions to improve access to and quality of health care, and improved access to self-care are needed. New interventions should be rigorously evaluated, consider outcomes beyond self-reported pain, and include placebo or sham treatments to address social desirability and placebo effects in effect estimates.

### Access to a supportive social environment

Interventions to combat stigma surrounding menstruation and provide a more supportive social environment are scarce in the region and globally.^9^ This is often assumed to be addressed as a by-product of educational interventions for students, yet no studies have tested this assumption and globally interventions have not reported leveraging relevant social, psychological, and behavioural theories in intervention design.

Findings from the review of progress towards MH in East Asia and Pacific^9^ and elsewhere,^63^ highlight the pervasive nature of menstrual-related stigma and its wide-ranging impacts. There is a need to develop theory-informed interventions to address the social environment surrounding menstruation; for example, interventions to dismantle harmful norms and social restrictions. Appropriate measures of social norms, and the social environment are needed to support evaluation of such interventions.^58^ Moreover, the influence of the social environment on the effectiveness of interventions addressing other MH requirements such as education or care seeking for disorders, should be captured to enable comparability and assessments of generalisability.

### Strengths and limitations

Our review was limited by searching undertaken only in English, potentially missing any studies in other languages. Due to the use of English in the title, one Chinese language study was identified and translated for inclusion. Interventions addressing care for MH discomfort and disorders presented challenges in determining eligibility. Clinical trials of pharmaceutical pain medication or homeopathic treatments were beyond the scope of this review. We included studies testing self-care strategies such as stretching and education interventions, however these focused only on the effectiveness of these strategies for pain reduction not for improving access to care. It is plausible some self-administrable pain care interventions were missed by our searching strategy and inclusion criteria. Strengths of our review included the adoption of the definition of MH as a framework to categorise interventions, enabling a detailed insight into evidence gaps and necessary next steps. Further, undertaking this review in the context of a regional review of policy and practice offered unique opportunities to contextualise findings.

### Implications for research and practice

Our review highlights a region-wide paucity of and urgent need for evidence to inform MH interventions. In the absence of evidence, practitioners and policy makers must ensure programmes are implemented with strong monitoring and evaluation frameworks; to detect potential harms and to inform improvements. Rigorous research is urgently needed to test the effectiveness of policy and programmes to support MH. We found that evaluations undertaken to date have not tested interventions aligned with current policy and practice efforts for example, interventions providing menstrual materials, and WASH services for MH are more complex than those that have been evaluated. Greater partnership between government, practitioners, and researchers is needed to evaluate practice-relevant interventions. Such trials or policy evaluations must comprehensively assess impacts of interventions on MH and on broader health and education outcomes that are hypothesised to be addressed by such programs.

Few studies and interventions were identified to address the supportive social environment for MH, or to improve healthcare seeking or access to care and self-care for menstrual discomforts and disorders. Co-development of interventions, alongside MH experts, clinicians, and communities is needed.

Further, included studies were mostly undertaken with adolescent girls in schools. This reflects the focus of most current focus of policy and programmes,^9^ but does not inform support to unmet menstrual needs of other demographic groups. Future research must target other demographic groups such as older people experiencing menopause or men and boys. Greater evidence for varying age groups and genders would help to inform policy and programming that addresses their specific needs.

Future research must clearly define intended intervention outcomes and adopt validated measurement tools to test them. Interventions must have a theory of change, informed by qualitative and quantitative research to understand MH, to identify desired outcomes, measure progress, and enable high quality programme evaluation. Practitioners implementing programmes, should utilise the MH definition and findings from this review to inform programmatic activities and incorporate MH requirements that are currently lacking, and seek to reach populations beyond girls in schools, while building in strong monitoring and evaluation plans and frameworks.

## Contributors

JH CH BY conceived the study. JH AH designed the methodology. AH CH PC HS undertook data collection. AH JH undertook analysis. All authors supported interpretation of findings. AH JH drafted the manuscript. All authors contributed to critical review of the manuscript. All authors have approved the manuscript.

## Data sharing statement

All data is contained within the manuscript and its supporting files.

## Declaration of interests

All authors declare no conflicts of interest.
