# Supplementary material for: Systematic review of the effectiveness of menstrual health interventions in low- and middle-income countries in the East Asia and Pacific region
Source: Lancet Reg Health Southeast Asia. 2023 Oct 19;20:100295. doi: 10.1016/j.lansea.2023.100295 (PMC10794107; doi:10.1016/j.lansea.2023.100295)
Supplement: Supplementary Materials 1–6 [file mmc1.pdf]

Supplementary Materials

Table of Contents

Supplementary Materials 1: PRISMA Checklist	2-3
Supplementary Materials 2: Full structured searching strategies for each database	4-14
Supplementary Materials 3: Full Text Screening - Excluded Studies	15-16
Supplementary Materials 4: Data extraction form	17-18
Supplementary Materials 5: Risk of Bias Assessment	19-20
Supplementary Materials 6: Summary of study findings	21-26

Supplementary Materials 1: PRISMA Checklist

Section and Topic	Item #	Checklist item	Location where item is reported
**TITLE**			
Title	1	Identify the report as a systematic review.	Title
**ABSTRACT**			
Abstract	2	See the PRISMA 2020 for Abstracts checklist.	Abstract
**INTRODUCTION**			
Rationale	3	Describe the rationale for the review in the context of existing knowledge.	Introduction, paragraphs 3-4
Objectives	4	Provide an explicit statement of the objective(s) or question(s) the review addresses.	Introduction, paragraph 3
**METHODS**			
Eligibility criteria	5	Specify the inclusion and exclusion criteria for the review and how studies were grouped for the syntheses.	Eligibility criteria
Information sources	6	Specify all databases, registers, websites, organisations, reference lists and other sources searched or consulted to identify studies. Specify the date when each source was last searched or consulted.	Eligibility criteria, Interventions
Search strategy	7	Present the full search strategies for all databases, registers and websites, including any filters and limits used.	Search methods. Supplementary materials 2
Selection process	8	Specify the methods used to decide whether a study met the inclusion criteria of the review, including how many reviewers screened each record and each report retrieved, whether they worked independently, and if applicable, details of automation tools used in the process.	Data collection and extraction
Data collection process	9	Specify the methods used to collect data from reports, including how many reviewers collected data from each report, whether they worked independently, any processes for obtaining or confirming data from study investigators, and if applicable, details of automation tools used in the process.	Data collection and extraction
Data items	10a	List and define all outcomes for which data were sought. Specify whether all results that were compatible with each outcome domain in each study were sought (e.g. for all measures, time points, analyses), and if not, the methods used to decide which results to collect.	Data collection and extraction
	10b	List and define all other variables for which data were sought (e.g. participant and intervention characteristics, funding sources). Describe any assumptions made about any missing or unclear information.	Data collection and extraction
Study risk of bias assessment	11	Specify the methods used to assess risk of bias in the included studies, including details of the tool(s) used, how many reviewers assessed each study and whether they worked independently, and if applicable, details of automation tools used in the process.	Risk of bias assessment
Effect measures	12	Specify for each outcome the effect measure(s) (e.g. risk ratio, mean difference) used in the synthesis or presentation of results.	N/A
Synthesis methods	13a	Describe the processes used to decide which studies were eligible for each synthesis (e.g. tabulating the study intervention characteristics and comparing against the planned groups for each synthesis (item #5)).	Synthesis methods
	13b	Describe any methods required to prepare the data for presentation or synthesis, such as handling of missing summary statistics, or data conversions.	N/A
	13c	Describe any methods used to tabulate or visually display results of individual studies and syntheses.	N/A
	13d	Describe any methods used to synthesize results and provide a rationale for the choice(s). If meta-analysis was performed, describe the model(s), method(s) to identify the presence and extent of statistical heterogeneity, and software package(s) used.	N/A
	13e	Describe any methods used to explore possible causes of heterogeneity among study results (e.g. subgroup analysis, meta-regression).	N/A
	13f	Describe any sensitivity analyses conducted to assess robustness of the synthesized results.	N/A
Reporting bias assessment	14	Describe any methods used to assess risk of bias due to missing results in a synthesis (arising from reporting biases).	N/A
Certainty assessment	15	Describe any methods used to assess certainty (or confidence) in the body of evidence for an outcome.	N/A
**RESULTS**			
Study selection	16a	Describe the results of the search and selection process, from the number of records identified in the search to the number of studies included in the review, ideally using a flow diagram.	Figure 1
	16b	Cite studies that might appear to meet the inclusion criteria, but which were excluded, and explain why they were excluded.	Supplementary Materials 4: Excluded studies
Study characteristics	17	Cite each included study and present its characteristics.	Table 2

Risk of bias in studies	18	Present assessments of risk of bias for each included study.	Figure 2. Supplementary materials 3.
Results of individual studies	19	For all outcomes, present, for each study: (a) summary statistics for each group (where appropriate) and (b) an effect estimate and its precision (e.g. confidence/credible interval), ideally using structured tables or plots.	N/A
Results of syntheses	20a	For each synthesis, briefly summarise the characteristics and risk of bias among contributing studies.	Results, Information and education through to Discussion
	20b	Present results of all statistical syntheses conducted. If meta-analysis was done, present for each the summary estimate and its precision (e.g. confidence/credible interval) and measures of statistical heterogeneity. If comparing groups, describe the direction of the effect.	N/A
	20c	Present results of all investigations of possible causes of heterogeneity among study results.	N/A
	20d	Present results of all sensitivity analyses conducted to assess the robustness of the synthesized results.	N/A
Reporting biases	21	Present assessments of risk of bias due to missing results (arising from reporting biases) for each synthesis assessed.	N/A
Certainty of evidence	22	Present assessments of certainty (or confidence) in the body of evidence for each outcome assessed.	N/A
**DISCUSSION**			
Discussion	23a	Provide a general interpretation of the results in the context of other evidence.	Discussion, Information and education, through to strengths and limitations
	23b	Discuss any limitations of the evidence included in the review.	Discussion, Strengths and limitations
	23c	Discuss any limitations of the review processes used.	Discussion, Strengths and limitations
	23d	Discuss implications of the results for practice, policy, and future research.	Discussion, Implications for research and practice
**OTHER INFORMATION**			
Registration and protocol	24a	Provide registration information for the review, including register name and registration number, or state that the review was not registered.	Methods, paragraph 1
	24b	Indicate where the review protocol can be accessed, or state that a protocol was not prepared.	Methods, paragraph 1
	24c	Describe and explain any amendments to information provided at registration or in the protocol.	Search methods, database searches.
Support	25	Describe sources of financial or non-financial support for the review, and the role of the funders or sponsors in the review.	Declarations, funding
Competing interests	26	Declare any competing interests of review authors.	Declarations, competing interests
Availability of data, code and other materials	27	Report which of the following are publicly available and where they can be found: template data collection forms; data extracted from included studies; data used for all analyses; analytic code; any other materials used in the review.	Search strategy, data extraction form, analytic code

From: Page MJ, McKenzie JE, Bossuyt PM, Boutron I, Hoffmann TC, Mulrow CD, et al. The PRISMA 2020 statement: an updated guideline for reporting systematic reviews. BMJ 2021;372:n71. doi: 10.1136/bmj.n71

Supplementary Materials 2: Search Strategy

Medline

#	Search term
1	Menstruation/
2	(menstru* or menses or catamenia or menarche or dysmenor* or endometrios?s or amenor*ea or menor*agi* or oligomenor* or premenstrual syndrome).mp.
3	(menst* adj3 (period or cycle or disorder* or pain*)).mp.
4	(period? adj1 (pain* or disorder* or irregular* or infrequent or abnormal)).mp.
5	(heavy period? or light period* or period discomfort*).mp.
6	1 or 2 or 3 or 4 or 5
7	(Pacific* or Melanesia* or Micronesia* or Polynesia* or Southeast* Asia or South East Asia or Indochin* Peninsula or (Fiji* or Ba or Bua or Cakaudrove or Kadavu or Lau or Lomaiviti or Macuata or Nadroga Navosa or Naitasiri or Namosi or Ra or Rewa or Serua or Tailevu or Rotuma) or (Yap or Chuuk or Pohnpei or Kosrae) or (Kiribati or Gilbert Islands or Phoenix Islands or Line Islands or Gilbertese)).mp.
8	(Papua New Guinea* or Papua Niugini* or Papua Niu Gini* or (Samoa* or Savaii or Upolu or Navigator island* or Tuamasaga or Aana or "Aiga i le Tai" or Atua or "Vaa o Fonoti" or Faasaleleaga or Gagaemauga or Gagaifomauga or Vaisigano or Satupaitea or Palauli or Afega or Leulumoega or Mulifanua or Lufilufi or Samamea or Safotulafai or Saleaula or Aopo or Asau or Satupaitea or Vailoa or Apia or Salelologa) or (Solomon Island* or Central Province or Choiseul or Guadalcanal or Isabel or Makira Ulawa or Malaita or "Rennell and Bellona" or Temotu or Capital Territory) or (Vanuatu* or "New Hebride*" or Malampa or Penama or Sanma or Shefa or Tafea or Torba)).mp.
9	(Cambodia* or Kampuchea* or khmer republic or Banteay Meanchey or Battambang or Kampong Cham or Kampong Chhnang or Kampong Speu or Kampong or Thom or Kampot or Kandal or Koh Kong or Kratie or Mondulkiri or Phnom Penh or Preah Vihear or Prey or Veng or Pursat or Ratanakiri or Siem Reap or Preah Sihanouk or Stung Treng or Svay Rieng or Takeo or Oddar or Meanchey or Kep or Pailin or Tboung Khmum or (Indonesia* or Celebes or East Indies or Aceh or Bangka Belitung Island* or Bengkulu or Jambi or Lampung or Sumatra* or Riau or Banten or Java* or Jakarta* or Yogyakarta* or Kalimantan* or Bali* or Nusa Tenggara* or Sulawesi* or Gorontalo* or Maluku* or Papua or West New Guinea or Irian Jaya or Madoera or Madura or Malay* or West Irian)).mp.
10	(Laos or laotian or Attapeu province* or Bokeo province* or Bolikhamxai province* or Champasak province* or Houaphanh province* or Khammouane or Luang Namtha province* or Luang Prabang province* or Oudomxay province* or Phongsaly province* or Salavan province* or Savannakhet province* or Vientiane province* or Sainyabuli province* or Sekong province* or Xaisomboun province* or Xiangkhouang province* or (Mongolia* or Arkhangai or Bayankhongor or Bayan Olgii or Darkhan Uul Dornod or Dornogovi or Dundgovi or Govi Altai or Khentii or Govisumber or Khovd or Khovsgol or Moron or Orkhon or mnogovi or Ovorkhangai or Selenge or Sukhbaatar or Tov or Uvs or Zavkhan or Ulaanbaatar) or (Burma or burmese or myanma or Myanmar or Yangon or Mandalay* or Magway* or Sagaing or Bago or Ayeyarwady or Tanintharyi or Kachin or Kayah or Kayin or Chin or Mon or Rakhine or Shan)).mp.
11	(Philippine* or filipin* or National Capital Region or Cordillera Administrative or Ilocos or Cagayan or Calabarzon or Southwestern Tagalog or Bicol or Western Visayas or Central Visayas or Eastern Visayas or Zamboanga or Northern Mindanao or Davao or Soccsksargen or Caraga or Bangsamoro or Cavite or Bulacan or Laguna or Rizal or Cebu or Pangasinan or Batangas or Negros Occidental or Pampanga or Nueva Ecija or Iloilo or Quezon or Camarines Sur or Isabela or Leyte or Bukidnon or Tarlac or Cotabato or Negros Oriental or Bohol or Albay or Maguindanao or Cagayan or "Lanao del Sur" or "Davao del Norte" or "Zamboanga del Sur" or "Zamboanga del Norte" or Sulu or South Cotabato or Misamis Oriental or Palawan or Masbate or Oriental Mindoro or Sultan Kudarat or Bataan or Sorsogon or "La Union" or Capiz or Cotabato City or Butuan or Koronadal or Davao City or "Cagayan de Oro" or Pagadian or Tacloban or Iloilo or Legazpi or Calapan or Calamba or "San Fernando" or Tuguegarao or Baguio or Manila or Quezon City or Caloocan or Zamboanga City or Cebu City or Taguig or Pasig or Valenzuela or General Santos or Paranaque or (Timor* or Bobonaro or Liquica or Dili or Baucau or Manatuto or Lautem or Cova Lima or Ainaro or Manufahi or Viqueque or Ermera or Aileu or Oecussi Ambeno)).mp.
12	(Vietnam* or Viet Nam* or Dong Bac Bo or Tay Bac Bo or Red River Delta or Dong Bang Song Hong or North Central Coast or Ha Noi Kinh or South Central Coast or Bac Trung Bo or Central Highlands or Duyen Hai Nam Trung Bo or Tay Nguyen or Southeast or Dong Nam Bo or Mien Dong or Mekong River Delta or Dong Bang Song Cuu Long or Tay Nam Bo Mien Tay or Bac Giang or Phu Tho or Quang Ninh or Thai Nguyen or Ha Tay or Hai Duoong or Hung Yen or Nam Dinh or Thai Binh or Vinh Phuc or Ha No or Hai Phong or Ha Tinh or Nghe An or Thanh Hoa or Thua Thien Hue or Binh dinh or Khanh Hoa or Quang Nam or Quang Ngai or Binh Thuan or Dak Lak or Gia Lai or Lam Dong or Dong Nai or Ho Chi Minh City or An Giang or Ben Tre or Ca Mau or Dong Thap or Kien Giang or Long An or Soc Trang or Tien Giang or Vinh Long or Can Tho or Viet Tri or Ha Long or Thai Nguyen or Ha Dong or Hai Duong or Hung Yen or Nam Dinh or Thai Binh or Vinh Yen or Ha Tinh or Vinh or Thanh Hoa or Hue or Qui Nhon or Nha Trang or Tam Ky or Quang Ngai or Phan Thiet or Buon Ma Thuot or Pleiku or Da Lat or Bien Hoa or Long Xuyen or Ben Tre or Ca Mau or Cao Lanh or Rach Gia or Tan An or Soc Trang or My Tho or Tra Vinh or Vinh Long).mp.
13	(China or Chinese or Zhongguo or Zhong gou* or Hong Kong or Macau or Beijing or Tianjin or Hebei or Liaoning or Shanghai or Jiangsu or Zhejiang or Fujian or Shandong or Guangdong or Hainan or Shanxi or Jilin or Heilongjiang or Anhui or Jiangxi or Henan or Hubei or Hunan or Inner Mongolia or Guangxi or Chongqing or Sichuan or Guizhou or Yunnan or Tibet or Shaanxi or Gansu or Qinghai or Ningxia or Xinjiang or (Korea* or Chagang or Hamgyong or Hwanghae or Kangwon or Pyongan or Ryanggang) or (malay* or Kuala Lumpur or Johor or Kedah or Kelantan or Malacca or Negeri Sembilan or Pahang or Penang or Perak or Perlis or Sabah or Sarawak or Selangor or Terengganu)).mp.
14	(Thai* or Siam or Bangkok or Amnat Charoen or Ang Thong or Bueng Kan or Buriram or Chachoengasao or Chai Nat or Chaiyaphum or Chanthaburi or Chiang Mai or Chiang Mai or Chiang Rai or Chonburi or Chumphon or Kalasin or Kamphaeng Phet or Kanchanaburi or Khon Kaen or Krabi or Lampang or Lamphun or Loei or Lopburi or Mae Hong Son or Maha Sarakham or Mukdahan or Nakhon Nayok or Nakhon Pathom or Nakhon Phanom or Nakhon Ratchasima or Nakhon Sawan or Nakhon Thammarat or Nan or Narathiwat or Nong Bua Lamphu or Nong Khai or Nonthaburi or Pathum Thani or Pattani or Phang Nga or Phatthalung or Phayao or Phetchabun or Phetchaburi or Phichit or Phitsanulok or Phra Nakhon Si Ayutthaya or Phrae or Phuket or Prachinburi or Prachuap Khiri Khan or Ranong or Ratchaburi or Rayong or Roi Et or Sa Kaeo or Sakon Nakhon or Samut Prakan or Samut Sakjon or Samut Songkhram or Saraburi or Satun or Sing Buri or Sisaket or

	Songkhla or Sukhothai or Suphan Buri or Surat Thani or Surin or Tak or Trang or Trat or Ubon Ratchathani or Udon Thani or Uthai Thani or Uttaradit or Yala or Yasothon).mp.
15	7 or 8 or 9 or 10 or 11 or 12 or 13 or 14
16	6 and 15
17	controlled clinical trial/ or randomized controlled trial/
18	cohort studies/ or controlled before-after studies/
19	(randomi?ed controlled trial or controlled trial or control trial or controlled study or control group? or experimental study or non-randomi?ed trial or pilot trial or randomi?e? or trial or randomly).mp.
20	(cohort or controlled before-after or "before-after study" or pre-post evaluation* or endline evaluation* or endline or “difference-in-differences” or pretest or posttest).mp.
21	(Evaluation OR comparison).mp.
22	(Program* OR policy OR intervention).mp.
23	17 or 18 or 19 or 20 or 21 or 22
24	16 and 23
25	limit 24 to yr="2015 -Current"

PsychINFO

#	Search term
1	Menstruation/
2	(menstru* or menses or catamenia or menarche or dysmenor* or endometrios?s or amenor*ea or menor*agi* or oligomenor* or premenstrual syndrome).mp.
3	(menst* adj3 (period or cycle or disorder* or pain*)).mp.
4	(period? adj1 (pain* or disorder* or irregular* or infrequent or abnormal)).mp.
5	(heavy period? or light period* or period discomfort*).mp.
6	1 or 2 or 3 or 4 or 5
7	(Pacific* or Melanesia* or Micronesia* or Polynesia* or Southeast* Asia or South East Asia or Indochin* Peninsula or (Fiji* or Ba or Bua or Cakaudrove or Kadavu or Lau or Lomaiviti or Macuata or Nadroga Navosa or Naitasiri or Namosi or Ra or Rewa or Serua or Tailevu or Rotuma) or (Yap or Chuuk or Pohnpei or Kosrae) or (Kiribati or Gilbert Islands or Phoenix Islands or Line Islands or Gilbertese)).mp.
8	(Papua New Guinea* or Papua Niugini* or Papua Niu Gini* or (Samoa* or Savaii or Upolu or Navigator island* or Tuamasaga or Aana or "Aiga i le Tai" or Atua or "Vaa o Fonoti" or Faasaleleaga or Gagaemauga or Gagaifomauga or Vaisigano or Satupaitea or Palauli or Afega or Leulumoega or Mulifanua or Lufilufi or Samamea or Safotulafai or Saleaula or Aopo or Asau or Satupaitea or Vailoa or Apia or Salelologa) or (Solomon Island* or Central Province or Choiseul or Guadalcanal or Isabel or Makira Ulawa or Malaita or "Rennell and Bellona" or Temotu or Capital Territory) or (Vanuatu* or "New Hebride*" or Malampa or Penama or Sanma or Shefa or Tafea or Torba)).mp.
9	(Cambodia* or Kampuchea* or khmer republic or Banteay Meanchey or Battambang or Kampong Cham or Kampong Chhnang or Kampong Speu or Kampong or Thom or Kampot or Kandal or Koh Kong or Kratie or Mondulkiri or Phnom Penh or Preah Vihear or Prey or Veng or Pursat or Ratanakiri or Siem Reap or Preah Sihanouk or Stung Treng or Svay Rieng or Takeo or Oddar or Meanchey or Kep or Pailin or Tboung Khmum or (Indonesia* or Celebes or East Indies or Aceh or Bangka Belitung Island* or Bengkulu or Jambi or Lampung or Sumatra* or Riau or Banten or Java* or Jakarta* or Yogyakarta* or Kalimantan* or Bali* or Nusa Tenggara* or Sulawesi* or Gorontalo* or Maluku* or Papua or West New Guinea or Irian Jaya or Madoera or Madura or Malay* or West Irian)).mp.
10	(Laos or laotian or Attapeu province* or Bokeo province* or Bolikhamxai province* or Champasak province* or Houaphanh province* or Khammouane or Luang Namtha province* or Luang Prabang province* or Oudomxay province* or Phongsaly province* or Salavan province* or Savannakhet province* or Vientiane province* or Sainyabuli province* or Sekong province* or Xaisomboun province* or Xiangkhouang province* or (Mongolia* or Arkhangai or Bayankhongor or Bayan Olgii or Darkhan Uul Dornod or Dornogovi or Dundgovi or Govi Altai or Khentii or Govisumber or Khovd or Khovsgol or Moron or Orkhon or mnogovi or Ovorkhangai or Selenge or Sukhbaatar or Tov or Uvs or Zavkhan or Ulaanbaatar) or (Burma or burmese or myanma or Myanmar or Yangon or Mandalay* or Magway* or Sagaing or Bago or Ayeyarwady or Tanintharyi or Kachin or Kayah or Kayin or Chin or Mon or Rakhine or Shan)).mp.
11	(Philippine* or filipin* or National Capital Region or Cordillera Administrative or Ilocos or Cagayan or Calabarzon or Southwestern Tagalog or Bicol or Western Visayas or Central Visayas or Eastern Visayas or Zamboanga or Northern Mindanao or Davao or Soccsksargen or Caraga or Bangsamoro or Cavite or Bulacan or Laguna or Rizal or Cebu or Pangasinan or Batangas or Negros Occidental or Pampanga or Nueva Ecija or Iloilo or Quezon or Camarines Sur or Isabela or Leyte or Bukidnon or Tarlac or Cotabato or Negros Oriental or Bohol or Albay or Maguindanao or Cagayan or "Lanao del Sur" or "Davao del Norte" or "Zamboanga del Sur" or "Zamboanga del Norte" or Sulu or South Cotabato or Misamis Oriental or Palawan or Masbate or Oriental Mindoro or Sultan Kudarat or Bataan or Sorsogon or "La Union" or Capiz or Cotabato City or Butuan or Koronadal or Davao City or "Cagayan de Oro" or Pagadian or Tacloban or Iloilo or Legazpi or Calapan or Calamba or "San Fernando" or Tuguegarao or Baguio or Manila or Quezon City or Caloocan or Zamboanga City or Cebu City or Taguig or Pasig or Valenzuela or General Santos or Paranaque or (Timor* or Bobonaro or Liquica or Dili or Baucau or Manatuto or Lautem or Cova Lima or Ainaro or Manufahi or Viqueque or Ermera or Aileu or Oecussi Ambeno)).mp.
12	(Vietnam* or Viet Nam* or Dong Bac Bo or Tay Bac Bo or Red River Delta or Dong Bang Song Hong or North Central Coast or Ha Noi Kinh or South Central Coast or Bac Trung Bo or Central Highlands or Duyen Hai Nam Trung Bo or Tay Nguyen or Southeast or Dong Nam Bo or Mien Dong or Mekong River Delta or Dong Bang Song Cuu Long or Tay Nam Bo Mien Tay or Bac Giang or Phu Tho or Quang Ninh or Thai Nguyen or Ha Tay or Hai Duoong or Hung Yen or Nam Dinh or Thai Binh or Vinh Phuc or Ha No or Hai Phong or Ha Tinh or Nghe An or Thanh Hoa or Thua Thien Hue or Binh dinh or Khanh Hoa or Quang Nam or Quang Ngai or Binh Thuan or Dak Lak or Gia Lai or Lam Dong or Dong Nai or Ho Chi Minh City or An Giang or Ben Tre or Ca Mau or Dong Thap or Kien Giang or Long An or Soc Trang or Tien Giang or Vinh Long or Can Tho or Viet Tri or Ha Long or Thai Nguyen or Ha Dong or Hai Duong or Hung Yen or Nam Dinh or Thai Binh or Vinh Yen or Ha Tinh or Vinh or Thanh Hoa or Hue or Qui Nhon or Nha Trang or Tam Ky or Quang Ngai or Phan

	Thiet or Buon Ma Thuot or Pleiku or Da Lat or Bien Hoa or Long Xuyen or Ben Tre or Ca Mau or Cao Lanh or Rach Gia or Tan An or Soc Trang or My Tho or Tra Vinh or Vinh Long).mp.
13	(China or Chinese or Zhongguo or Zhong gou* or Hong Kong or Macau or Beijing or Tianjin or Hebei or Liaoning or Shanghai or Jiangsu or Zhejiang or Fujian or Shandong or Guangdong or Hainan or Shanxi or Jilin or Heilongjiang or Anhui or Jiangxi or Henan or Hubei or Hunan or Inner Mongolia or Guangxi or Chongqing or Sichuan or Guizhou or Yunnan or Tibet or Shaanxi or Gansu or Qinghai or Ningxia or Xinjiang or (Korea* or Chagang or Hamgyong or Hwanghae or Kangwon or Pyongan or Ryanggang) or (malay* or Kuala Lumpur or Johor or Kedah or Kelantan or Malacca or Negeri Sembilan or Pahang or Penang or Perak or Perlis or Sabah or Sarawak or Selangor or Terengganu)).mp.
14	(Thai* or Siam or Bangkok or Amnat Charoen or Ang Thong or Bueng Kan or Buriram or Chachoengasao or Chai Nat or Chaiyaphum or Chanthaburi or Chiang Mai or Chiang Mai or Chiang Rai or Chonburi or Chumphon or Kalasin or Kamphaeng Phet or Kanchanaburi or Khon Kaen or Krabi or Lampang or Lamphun or Loei or Lopburi or Mae Hong Son or Maha Sarakham or Mukdahan or Nakhon Nayok or Nakhon Pathom or Nakhon Phanom or Nakhon Ratchasima or Nakhon Sawan or Nakhon Thammarat or Nan or Narathiwat or Nong Bua Lamphu or Nong Khai or Nonthaburi or Pathum Thani or Pattani or Phang Nga or Phatthalung or Phayao or Phetchabun or Phetchaburi or Phichit or Phitsanulok or Phra Nakhon Si Ayutthaya or Phrae or Phuket or Prachinburi or Prachuap Khiri Khan or Ranong or Ratchaburi or Rayong or Roi Et or Sa Kaeo or Sakon Nakhon or Samut Prakan or Samut Sakjon or Samut Songkhram or Saraburi or Satun or Sing Buri or Sisaket or Songkhla or Sukhothai or Suphan Buri or Surat Thani or Surin or Tak or Trang or Trat or Ubon Ratchathani or Udon Thani or Uthai Thani or Uttaradit or Yala or Yasothon).mp.
15	7 or 8 or 9 or 10 or 11 or 12 or 13 or 14
16	6 and 15
17	clinical trials/ or randomized controlled trials/
18	experimental design/ or evidence based practice/
19	Pretesting/ or posttesting/
20	Intervention/ or evaluation/ or needs assessment/ or program evaluation/
21	(randomi?ed controlled trial or controlled trial or control trial or controlled study or control group? or experimental study or non-randomi?ed trial or pilot trial or randomi?e? or trial or randomly).mp.
22	(cohort or controlled before-after or "before-after study" or pre-post evaluation* or endline evaluation* or endline or “difference-in-differences” or pretest or posttest).mp.
23	(Evaluation OR comparison).mp.
24	(Program* OR policy OR intervention).mp.
25	17 or 18 or 19 or 20 or 21 or 22 or 23 or 24
26	16 and 25
26	limit 25 to yr="2015 -Current"

Embase

#	Search term
1	Menstruation/
2	(menstru* or menses or catamenia or menarche).mp.
3	(dysmenor* or endometrios?s or amenor*ea or menor*agi* or oligomenor* or premenstrual syndrome).mp.
4	(menst* adj3 (period or cycle or disorder* or pain*)).mp.
5	(period? adj1 (pain* or disorder* or irregular* or infrequent or abnormal)).mp.
6	(heavy period? or light period* or period discomfort*).mp.
7	3 or 4 or 5 or 6
8	(Self-care or health care access).mp.
9	7 and 8
10	1 or 2 or 9
11	(Pacific* or Melanesia* or Micronesia* or Polynesia* or Southeast* Asia or South East Asia or Indochin* Peninsula or (Fiji* or Ba or Bua or Cakaudrove or Kadavu or Lau or Lomaiviti or Macuata or Nadroga Navosa or Naitasiri or Namosi or Ra or Rewa or Serua or Tailevu or Rotuma) or (Yap or Chuuk or Pohnpei or Kosrae) or (Kiribati or Gilbert Islands or Phoenix Islands or Line Islands or Gilbertese)).mp.
12	(Papua New Guinea* or Papua Niugini* or Papua Niu Gini* or (Samoa* or Savaii or Upolu or Navigator island* or Tuamasaga or Aana or "Aiga i le Tai" or Atua or "Vaa o Fonoti" or Faasaleleaga or Gagaemauga or Gagaifomauga or Vaisigano or Satupaitea or Palauli or Afega or Leulumoega or Mulifanua or Lufilufi or Samamea or Safotulafai or Saleaula or Aopo or Asau or Satupaitea or Vailoa or Apia or Salelologa) or (Solomon Island* or Central Province or Choiseul or Guadalcanal or Isabel or Makira Ulawa or Malaita or "Rennell and Bellona" or Temotu or Capital Territory) or (Vanuatu* or "New Hebride*" or Malampa or Penama or Sanma or Shefa or Tafea or Torba)).mp.
13	(Cambodia* or Kampuchea* or khmer republic or Banteay Meanchey or Battambang or Kampong Cham or Kampong Chhnang or Kampong Speu or Kampong or Thom or Kampot or Kandal or Koh Kong or Kratie or Mondulkiri or Phnom Penh or Preah Vihear or Prey or Veng or Pursat or Ratanakiri or Siem Reap or Preah Sihanouk or Stung Treng or Svay Rieng or Takeo or Oddar or Meanchey or Kep or Pailin or Tboung Khmum or (Indonesia* or Celebes or East Indies or Aceh or Bangka Belitung Island* or Bengkulu or Jambi or Lampung

	or Sumatra* or Riau or Banten or Java* or Jakarta* or Yogyakarta* or Kalimantan* or Bali* or Nusa Tenggara* or Sulawesi* or Gorontalo* or Maluku* or Papua or West New Guinea or Irian Jaya or Madoera or Madura or Malay* or West Irian)).mp.
14	(Laos or laotian or Attapeu province* or Bokeo province* or Bolikhamxai province* or Champasak province* or Houaphanh province* or Khammouane or Luang Namtha province* or Luang Prabang province* or Oudomxay province* or Phongsaly province* or Salavan province* or Savannakhet province* or Vientiane province* or Sainyabuli province* or Sekong province* or Xaisomboun province* or Xiangkhouang province* or (Mongolia* or Arkhangai or Bayankhongor or Bayan Olgii or Darkhan Uul Dornod or Dornogovi or Dundgovi or Govi Altai or Khentii or Govisumber or Khovd or Khovsgol or Moron or Orkhon or mnogovi or Ovorkhangai or Selenge or Sukhbaatar or Tov or Uvs or Zavkhan or Ulaanbaatar) or (Burma or burmese or myanma or Myanmar or Yangon or Mandalay* or Magway* or Sagaing or Bago or Ayeyarwady or Tanintharyi or Kachin or Kayah or Kayin or Chin or Mon or Rakhine or Shan)).mp.
15	(Philippine* or filipin* or National Capital Region or Cordillera Administrative or Ilocos or Cagayan or Calabarzon or Southwestern Tagalog or Bicol or Western Visayas or Central Visayas or Eastern Visayas or Zamboanga or Northern Mindanao or Davao or Soccsksargen or Caraga or Bangsamoro or Cavite or Bulacan or Laguna or Rizal or Cebu or Pangasinan or Batangas or Negros Occidental or Pampanga or Nueva Ecija or Iloilo or Quezon or Camarines Sur or Isabela or Leyte or Bukidnon or Tarlac or Cotabato or Negros Oriental or Bohol or Albay or Maguindanao or Cagayan or "Lanao del Sur" or "Davao del Norte" or "Zamboanga del Sur" or "Zamboanga del Norte" or Sulu or South Cotabato or Misamis Oriental or Palawan or Masbate or Oriental Mindoro or Sultan Kudarat or Bataan or Sorsogon or "La Union" or Capiz or Cotabato City or Butuan or Koronadal or Davao City or "Cagayan de Oro" or Pagadian or Tacloban or Iloilo or Legazpi or Calapan or Calamba or "San Fernando" or Tuguegarao or Baguio or Manila or Quezon City or Caloocan or Zamboanga City or Cebu City or Taguig or Pasig or Valenzuela or General Santos or Paranaque or (Timor* or Bobonaro or Liquica or Dili or Baucau or Manatuto or Lautem or Cova Lima or Ainaro or Manufahi or Viqueque or Ermera or Aileu or Oecussi Ambeno)).mp.
16	(Vietnam* or Viet Nam* or Dong Bac Bo or Tay Bac Bo or Red River Delta or Dong Bang Song Hong or North Central Coast or Ha Noi Kinh or South Central Coast or Bac Trung Bo or Central Highlands or Duyen Hai Nam Trung Bo or Tay Nguyen or Southeast or Dong Nam Bo or Mien Dong or Mekong River Delta or Dong Bang Song Cuu Long or Tay Nam Bo Mien Tay or Bac Giang or Phu Tho or Quang Ninh or Thai Nguyen or Ha Tay or Hai Duoong or Hung Yen or Nam Dinh or Thai Binh or Vinh Phuc or Ha No or Hai Phong or Ha Tinh or Nghe An or Thanh Hoa or Thua Thien Hue or Binh dinh or Khanh Hoa or Quang Nam or Quang Ngai or Binh Thuan or Dak Lak or Gia Lai or Lam Dong or Dong Nai or Ho Chi Minh City or An Giang or Ben Tre or Ca Mau or Dong Thap or Kien Giang or Long An or Soc Trang or Tien Giang or Vinh Long or Can Tho or Viet Tri or Ha Long or Thai Nguyen or Ha Dong or Hai Duong or Hung Yen or Nam Dinh or Thai Binh or Vinh Yen or Ha Tinh or Vinh or Thanh Hoa or Hue or Qui Nhon or Nha Trang or Tam Ky or Quang Ngai or Phan Thiet or Buon Ma Thuot or Pleiku or Da Lat or Bien Hoa or Long Xuyen or Ben Tre or Ca Mau or Cao Lanh or Rach Gia or Tan An or Soc Trang or My Tho or Tra Vinh or Vinh Long).mp.
17	(China or Chinese or Zhongguo or Zhong gou* or Hong Kong or Macau or Beijing or Tianjin or Hebei or Liaoning or Shanghai or Jiangsu or Zhejiang or Fujian or Shandong or Guangdong or Hainan or Shanxi or Jilin or Heilongjiang or Anhui or Jiangxi or Henan or Hubei or Hunan or Inner Mongolia or Guangxi or Chongqing or Sichuan or Guizhou or Yunnan or Tibet or Shaanxi or Gansu or Qinghai or Ningxia or Xinjiang or (Korea* or Chagang or Hamgyong or Hwanghae or Kangwon or Pyongan or Ryanggang) or (malay* or Kuala Lumpur or Johor or Kedah or Kelantan or Malacca or Negeri Sembilan or Pahang or Penang or Perak or Perlis or Sabah or Sarawak or Selangor or Terengganu)).mp.
18	(Thai* or Siam or Bangkok or Amnat Charoen or Ang Thong or Bueng Kan or Buriram or Chachoengasao or Chai Nat or Chaiyaphum or Chanthaburi or Chiang Mai or Chiang Mai or Chiang Rai or Chonburi or Chumphon or Kalasin or Kamphaeng Phet or Kanchanaburi or Khon Kaen or Krabi or Lampang or Lamphun or Loei or Lopburi or Mae Hong Son or Maha Sarakham or Mukdahan or Nakhon Nayok or Nakhon Pathom or Nakhon Phanom or Nakhon Ratchasima or Nakhon Sawan or Nakhon Thammarat or Nan or Narathiwat or Nong Bua Lamphu or Nong Khai or Nonthaburi or Pathum Thani or Pattani or Phang Nga or Phatthalung or Phayao or Phetchabun or Phetchaburi or Phichit or Phitsanulok or Phra Nakhon Si Ayutthaya or Phrae or Phuket or Prachinburi or Prachuap Khiri Khan or Ranong or Ratchaburi or Rayong or Roi Et or Sa Kaeo or Sakon Nakhon or Samut Prakan or Samut Sakjon or Samut Songkhram or Saraburi or Satun or Sing Buri or Sisaket or Songkhla or Sukhothai or Suphan Buri or Surat Thani or Surin or Tak or Trang or Trat or Ubon Ratchathani or Udon Thani or Uthai Thani or Uttaradit or Yala or Yasothon).mp.
19	11 or 12 or 13 or 14 or 15 or 16 or 17 or 18
20	10 and 19
21	controlled clinical trial/ or "controlled clinical trial (topic)"/ or "clinical trial (topic)"/ or methodology/ or randomized controlled trial/ or "randomized controlled trial (topic)"/ or randomization/
22	experimental design/ or cohort analysis/ or epidemiology/ or pretest posttest design/
23	evaluation study/ or intervention study/ or program evaluation/
24	(randomi?ed controlled trial or controlled trial or control trial or controlled study or control group? or experimental study or non-randomi?ed trial or pilot trial or trial).mp.
25	(cohort or controlled before-after or "before-after study" or pre-post evaluation* or endline evaluation* or endline or difference-in-differences or pretest or posttest).mp.
26	21 or 22 or 23 or 24 or 25
27	20 and 26
28	limit 27 to yr="2015 -Current"

Cochrane Central Register of Controlled Trials (CENTRAL)

	**Search term**
1	Menstruation/
2	(menstru* or menses or catamenia or menarche) or dysmenor* or endometrios?s or amenor*ea or menor*agi* or oligomenor* or premenstrual syndrome).ti,ab,kw
3	(menst* adj3 (period or cycle or disorder* or pain*)).ti,ab,kw
4	(period? adj1 (pain* or disorder* or irregular* or infrequent or abnormal)).ti,ab,kw

5	(heavy period? or light period* or period discomfort*).ti,ab,kw
6	1 or 2 or 3 or 4 or 5
7	(Pacific* or Melanesia* or Micronesia* or Polynesia* or Southeast* Asia or South East Asia or Indochin* Peninsula or (Fiji* or Ba or Bua or Cakaudrove or Kadavu or Lau or Lomaiviti or Macuata or Nadroga Navosa or Naitasiri or Namosi or Ra or Rewa or Serua or Tailevu or Rotuma) or (Yap or Chuuk or Pohnpei or Kosrae) or (Kiribati or Gilbert Islands or Phoenix Islands or Line Islands or Gilbertese)).mp.
8	(Papua New Guinea* or Papua Niugini* or Papua Niu Gini* or (Samoa* or Savaii or Upolu or Navigator island* or Tuamasaga or Aana or "Aiga i le Tai" or Atua or "Vaa o Fonoti" or Faasaleleaga or Gagaemauga or Gagaifomauga or Vaisigano or Satupaitea or Palauli or Afega or Leulumoega or Mulifanua or Lufilufi or Samamea or Safotulafai or Saleaula or Aopo or Asau or Satupaitea or Vailoa or Apia or Salelologa) or (Solomon Island* or Central Province or Choiseul or Guadalcanal or Isabel or Makira Ulawa or Malaita or "Rennell and Bellona" or Temotu or Capital Territory) or (Vanuatu* or "New Hebride*" or Malampa or Penama or Sanma or Shefa or Tafea or Torba)).mp.
9	(Cambodia* or Kampuchea* or khmer republic or Banteay Meanchey or Battambang or Kampong Cham or Kampong Chhnang or Kampong Speu or Kampong or Thom or Kampot or Kandal or Koh Kong or Kratie or Mondulkiri or Phnom Penh or Preah Vihear or Prey or Veng or Pursat or Ratanakiri or Siem Reap or Preah Sihanouk or Stung Treng or Svay Rieng or Takeo or Oddar or Meanchey or Kep or Pailin or Tboung Khmum or (Indonesia* or Celebes or East Indies or Aceh or Bangka Belitung Island* or Bengkulu or Jambi or Lampung or Sumatra* or Riau or Banten or Java* or Jakarta* or Yogyakarta* or Kalimantan* or Bali* or Nusa Tenggara* or Sulawesi* or Gorontalo* or Maluku* or Papua or West New Guinea or Irian Jaya or Madoera or Madura or Malay* or West Irian)).mp.
10	(Laos or laotian or Attapeu province* or Bokeo province* or Bolikhamxai province* or Champasak province* or Houaphanh province* or Khammouane or Luang Namtha province* or Luang Prabang province* or Oudomxay province* or Phongsaly province* or Salavan province* or Savannakhet province* or Vientiane province* or Sainyabuli province* or Sekong province* or Xaisomboun province* or Xiangkhouang province* or (Mongolia* or Arkhangai or Bayankhongor or Bayan Olgii or Darkhan Uul Dornod or Dornogovi or Dundgovi or Govi Altai or Khentii or Govisumber or Khovd or Khovsgol or Moron or Orkhon or mnogovi or Ovorkhangai or Selenge or Sukhbaatar or Tov or Uvs or Zavkhan or Ulaanbaatar) or (Burma or burmese or myanma or Myanmar or Yangon or Mandalay* or Magway* or Sagaing or Bago or Ayeyarwady or Tanintharyi or Kachin or Kayah or Kayin or Chin or Mon or Rakhine or Shan)).mp.
11	(Philippine* or filipin* or National Capital Region or Cordillera Administrative or Ilocos or Cagayan or Calabarzon or Southwestern Tagalog or Bicol or Western Visayas or Central Visayas or Eastern Visayas or Zamboanga or Northern Mindanao or Davao or Soccsksargen or Caraga or Bangsamoro or Cavite or Bulacan or Laguna or Rizal or Cebu or Pangasinan or Batangas or Negros Occidental or Pampanga or Nueva Ecija or Iloilo or Quezon or Camarines Sur or Isabela or Leyte or Bukidnon or Tarlac or Cotabato or Negros Oriental or Bohol or Albay or Maguindanao or Cagayan or "Lanao del Sur" or "Davao del Norte" or "Zamboanga del Sur" or "Zamboanga del Norte" or Sulu or South Cotabato or Misamis Oriental or Palawan or Masbate or Oriental Mindoro or Sultan Kudarat or Bataan or Sorsogon or "La Union" or Capiz or Cotabato City or Butuan or Koronadal or Davao City or "Cagayan de Oro" or Pagadian or Tacloban or Iloilo or Legazpi or Calapan or Calamba or "San Fernando" or Tuguegarao or Baguio or Manila or Quezon City or Caloocan or Zamboanga City or Cebu City or Taguig or Pasig or Valenzuela or General Santos or Paranaque or (Timor* or Bobonaro or Liquica or Dili or Baucau or Manatuto or Lautem or Cova Lima or Ainaro or Manufahi or Viqueque or Ermera or Aileu or Oecussi Ambeno)).mp.
12	(Vietnam* or Viet Nam* or Dong Bac Bo or Tay Bac Bo or Red River Delta or Dong Bang Song Hong or North Central Coast or Ha Noi Kinh or South Central Coast or Bac Trung Bo or Central Highlands or Duyen Hai Nam Trung Bo or Tay Nguyen or Southeast or Dong Nam Bo or Mien Dong or Mekong River Delta or Dong Bang Song Cuu Long or Tay Nam Bo Mien Tay or Bac Giang or Phu Tho or Quang Ninh or Thai Nguyen or Ha Tay or Hai Duoong or Hung Yen or Nam Dinh or Thai Binh or Vinh Phuc or Ha No or Hai Phong or Ha Tinh or Nghe An or Thanh Hoa or Thua Thien Hue or Binh dinh or Khanh Hoa or Quang Nam or Quang Ngai or Binh Thuan or Dak Lak or Gia Lai or Lam Dong or Dong Nai or Ho Chi Minh City or An Giang or Ben Tre or Ca Mau or Dong Thap or Kien Giang or Long An or Soc Trang or Tien Giang or Vinh Long or Can Tho or Viet Tri or Ha Long or Thai Nguyen or Ha Dong or Hai Duong or Hung Yen or Nam Dinh or Thai Binh or Vinh Yen or Ha Tinh or Vinh or Thanh Hoa or Hue or Qui Nhon or Nha Trang or Tam Ky or Quang Ngai or Phan Thiet or Buon Ma Thuot or Pleiku or Da Lat or Bien Hoa or Long Xuyen or Ben Tre or Ca Mau or Cao Lanh or Rach Gia or Tan An or Soc Trang or My Tho or Tra Vinh or Vinh Long).mp.
13	(China or Chinese or Zhongguo or Zhong gou* or Hong Kong or Macau or Beijing or Tianjin or Hebei or Liaoning or Shanghai or Jiangsu or Zhejiang or Fujian or Shandong or Guangdong or Hainan or Shanxi or Jilin or Heilongjiang or Anhui or Jiangxi or Henan or Hubei or Hunan or Inner Mongolia or Guangxi or Chongqing or Sichuan or Guizhou or Yunnan or Tibet or Shaanxi or Gansu or Qinghai or Ningxia or Xinjiang or (Korea* or Chagang or Hamgyong or Hwanghae or Kangwon or Pyongan or Ryanggang) or (malay* or Kuala Lumpur or Johor or Kedah or Kelantan or Malacca or Negeri Sembilan or Pahang or Penang or Perak or Perlis or Sabah or Sarawak or Selangor or Terengganu)).mp.
14	(Thai* or Siam or Bangkok or Amnat Charoen or Ang Thong or Bueng Kan or Buriram or Chachoengasao or Chai Nat or Chaiyaphum or Chanthaburi or Chiang Mai or Chiang Mai or Chiang Rai or Chonburi or Chumphon or Kalasin or Kamphaeng Phet or Kanchanaburi or Khon Kaen or Krabi or Lampang or Lamphun or Loei or Lopburi or Mae Hong Son or Maha Sarakham or Mukdahan or Nakhon Nayok or Nakhon Pathom or Nakhon Phanom or Nakhon Ratchasima or Nakhon Sawan or Nakhon Thammarat or Nan or Narathiwat or Nong Bua Lamphu or Nong Khai or Nonthaburi or Pathum Thani or Pattani or Phang Nga or Phatthalung or Phayao or Phetchabun or Phetchaburi or Phichit or Phitsanulok or Phra Nakhon Si Ayutthaya or Phrae or Phuket or Prachinburi or Prachuap Khiri Khan or Ranong or Ratchaburi or Rayong or Roi Et or Sa Kaeo or Sakon Nakhon or Samut Prakan or Samut Sakjon or Samut Songkhram or Saraburi or Satun or Sing Buri or Sisaket or Songkhla or Sukhothai or Suphan Buri or Surat Thani or Surin or Tak or Trang or Trat or Ubon Ratchathani or Udon Thani or Uthai Thani or Uttaradit or Yala or Yasothon).mp
15	7 or 8 or 9 or 10 or 11 or 12 or 13 or 14
16	6 and 15
17	controlled clinical trial/ or randomized controlled trial/
18	cohort studies/ or controlled before-after studies/
19	(“randomi?ed controlled trial” or “controlled trial” or “control trial” or “controlled study” or “control group?” or “experimental study” or “non-randomi?ed trial” or “pilot trial” or randomi?e? or trial or randomly).mp.
20	(cohort or “controlled before-after study” or "before-after study" or “pre-post evaluation*” or “endline evaluation*” or endline or “difference-in-differences” or pretest or posttest).mp.
21	(Evaluation OR comparison).mp.
22	(Program* OR policy OR intervention).mp.

23	17 or 18 or 19 or 20 or 21 or 22
24	16 and 23
25	limit 24 to yr="2015 -Current"

ASSIA (Applied Social Science Index and Abstracts) and PROQUEST Dissertation and Theses

	Search term
1	noft(menstru* or menses or catamenia or menarche)
2	noft(dysmenor* or endometrios?s or amenor*ea or menor*agi* or oligomenor* or “premenstrual syndrome”)
3	noft(menst* NEAR/3 (period or cycle or disorder* or pain*))
4	noft(period? NEAR/1 (pain* or disorder* or irregular* or infrequent or abnormal))
5	noft(“heavy period?” or “light period*” or “period discomfort*”)
6	S2 OR S3 OR S4 or S5
7	noft(Self-care or health care access)
8	S6 AND S7
9	S1 OR S8
10	noft(Pacific* or Melanesia* or Micronesia* or Polynesia* or “Southeast* Asia” or “South East Asia” or “Indochin* Peninsula” or (Fiji* or Ba or Bua or Cakaudrove or Kadavu or Lau or Lomaiviti or Macuata or “Nadroga Navosa” or Naitasiri or Namosi or Ra or Rewa or Serua or Tailevu or Rotuma) or (Yap or Chuuk or Pohnpei or Kosrae) or (Kiribati or “Gilbert Islands” or “Phoenix Islands” or “Line Islands” or Gilbertese))
11	noft(“Papua New Guinea*” or Papua Niugini* or “Papua Niu Gini*” or (Samoa* or Savaii or Upolu or “Navigator island*” or Tuamasaga or Aana or “Aiga i le Tai” or Atua or "Vaa o Fonoti" or Faasaleleaga or Gagaemauga or Gagaifomauga or Vaisigano or Satupaitea or Palauli or Afega or Leulumoega or Mulifanua or Lufilufi or Samamea or Safotulafai or Saleaula or Aopo or Asau or Satupaitea or Vailoa or Apia or Salelologa) or (“Solomon Island*” or “Central Province” or Choiseul or Guadalcanal or Isabel or “Makira Ulawa” or Malaita or "Rennell and Bellona" or Temotu or “Capital Territory”) or (Vanuatu* or "New Hebride*" or Malampa or Penama or Sanma or Shefa or Tafea or Torba))
12	noft(Cambodia* or Kampuchea* or “khmer republic” or “Banteay Meanchey” or Battambang or “Kampong Cham” or “Kampong Chhnang” or “Kampong Speu” or “Kampong Thom” or Kampot or Kandal or “Koh Kong” or Kratie or Mondulkiri or “Phnom Penh” or “Preah Vihear” or Prey or Veng or Pursat or Ratanakiri or “Siem Reap” or “Preah Sihanouk” or “Stung Treng” or “Svay Rieng” or Takeo or Oddar or Meanchey or Kep or Pailin or “Tboung Khmum” or (Indonesia* or Celebes or “East Indies” or Aceh or “Bangka Belitung Island*” or Bengkulu or Jambi or Lampung or Sumatra* or Riau or Banten or Java* or Jakarta* or Yogyakarta* or Kalimantan* or Bali* or “Nusa Tenggara*” or Sulawesi* or Gorontalo* or Maluku* or Papua or “West New Guinea” or “Irian Jaya” or Madoera or Madura or Malay* or West Irian))
13	noft(Laos or laotian or “Attapeu*” or “Bokeo*” or “Bolikhamxai*” or “Champasak*” or “Houaphanh*” or Khammouane or “Luang Namtha*” or “Luang Prabang*” or “Oudomxay*” or “Phongsaly*” or “Salavan*” or “Savannakhet*” or “Vientiane*” or “Sainyabuli*” or “Sekong*” or “Xaisomboun*” or “Xiangkhouang*” or (Mongolia* or Arkhangai or Bayankhongor or “Bayan Olgii” or “Darkhan Uul Dornod” or Dornogovi or Dundgovi or “Govi Altai” or Khentii or Govisumber or Khovd or Khovsgol or Moron or Orkhon or mnogovi or Ovorkhangai or Selenge or Sukhbaatar or Tov or Uvs or Zavkhan or Ulaanbaatar) or (Burma or burmese or myanma or Myanmar or Yangon or Mandalay* or Magway* or Sagaing or Bago or Ayeyarwady or Tanintharyi or Kachin or Kayah or Kayin or Chin or Mon or Rakhine or Shan))
14	noft(Philippine* or filipin* or “National Capital Region” or “Cordillera Administrative” or Ilocos or Cagayan or Calabarzon or “Southwestern Tagalog” or Bicol or Western Visayas or “Central Visayas” or “Eastern Visayas” or Zamboanga or “Northern Mindanao” or Davao or Soccsksargen or Caraga or Bangsamoro or Cavite or Bulacan or Laguna or Rizal or Cebu or Pangasinan or Batangas or “Negros Occidental” or Pampanga or “Nueva Ecija” or Iloilo or Quezon or “Camarines Sur” or Isabela or Leyte or Bukidnon or Tarlac or Cotabato or “Negros Oriental” or Bohol or Albay or Maguindanao or Cagayan or "Lanao del Sur" or "Davao del Norte" or "Zamboanga del Sur" or "Zamboanga del Norte" or Sulu or “South Cotabato” or “Misamis Oriental” or Palawan or Masbate or “Oriental Mindoro” or “Sultan Kudarat” or Bataan or Sorsogon or "La Union" or Capiz or “Cotabato City” or Butuan or Koronadal or “Davao City” or "Cagayan de Oro" or Pagadian or Tacloban or Iloilo or Legazpi or Calapan or Calamba or "San Fernando" or Tuguegarao or Baguio or Manila or “Quezon City” or Caloocan or “Zamboanga City” or “Cebu City” or Taguig or Pasig or Valenzuela or “General Santos” or Paranaque or (Timor* or Bobonaro or Liquica or Dili or Baucau or Manatuto or Lautem or “Cova Lima” or Ainaro or Manufahi or Viqueque or Ermera or Aileu or “Oecussi Ambeno”))
15	noft(Vietnam* or “Viet Nam*” or “Dong Bac Bo” or “Tay Bac Bo” or “Red River Delta” or “Dong Bang Song Hong” or “North Central Coast” or “Ha Noi Kinh” or “South Central Coast” or “Bac Trung Bo” or “Central Highlands” or “Duyen Hai Nam Trung Bo” or “Tay Nguyen” or “Southeast” or “Dong Nam Bo” or “Mien Dong” or “Mekong River Delta” or “Dong Bang Song Cuu Long” or “Tay Nam Bo Mien Tay” or “Bac Giang” or “Phu Tho” or “Quang Ninh” or “Thai Nguyen” or “Ha Tay” or “Hai Duoong” or “Hung Yen” or “Nam Dinh” or “Thai Binh” or “Vinh Phuc” or “Ha No” or “Hai Phong” or “Ha Tinh” or “Nghe An” or “Thanh Hoa” or “Thua Thien Hue” or “Binh dinh” or “Khanh Hoa” or “Quang Nam” or “Quang Ngai” or “Binh Thuan” or “Dak Lak” or “Gia Lai” or “Lam Dong” or “Dong Nai” or “Ho Chi Minh City” or “An Giang” or “Ben Tre” or “Ca Mau” or “Dong Thap” or “Kien Giang” or “Long An” or “Soc Trang” or “Tien Giang” or “Vinh Long” or “Can Tho” or “Viet Tri” or “Ha Long” or “Thai Nguyen” or “Ha Dong” or “Hai Duong” or “Hung Yen” or “Nam Dinh” or “Thai Binh” or “Vinh Yen” or “Ha Tinh” or Vinh or “Thanh Hoa” or Hue or “Qui Nhon” or “Nha Trang” or “Tam Ky” or “Quang Ngai” or “Phan Thiet” or “Buon Ma Thuot” or Pleiku or “Da Lat” or “Bien Hoa” or “Long Xuyen” or “Ben Tre” or “Ca Mau” or “Cao Lanh” or “Rach Gia” or “Tan An” or “Soc Trang” or “My Tho” or “Tra Vinh” or “Vinh Long”)
16	noft(China or Chinese or Zhongguo or “Zhong gou*” or “Hong Kong” or Macau or Beijing or Tianjin or Hebei or Liaoning or Shanghai or Jiangsu or Zhejiang or Fujian or Shandong or Guangdong or Hainan or Shanxi or Jilin or Heilongjiang or Anhui or Jiangxi or Henan or Hubei or Hunan or Inner Mongolia or Guangxi or Chongqing or Sichuan or Guizhou or Yunnan or Tibet or Shaanxi or Gansu or Qinghai or Ningxia or Xinjiang or (Korea* or Chagang or Hamgyong or Hwanghae or Kangwon or Pyongan or Ryanggang) or (malay* or “Kuala

	Lumpur" or Johor or Kedah or Kelantan or Malacca or "Negeri Sembilan" or Pahang or Penang or Perak or Perlis or Sabah or Sarawak or Selangor or Terengganu))
17	noft(Thai* or Siam or Bangkok or "Amnat Charoen" or "Ang Thong" or "Bueng Kan" or Buriram or Chachoengasao or "Chai Nat" or Chaiyaphum or Chanthaburi or Chiang Mai or "Chiang Mai" or "Chiang Rai" or Chonburi or Chumphon or Kalasin or "Kamphaeng Phet" or Kanchanaburi or "Khon Kaen" or Krabi or Lampang or Lamphun or Loei or Lopburi or "Mae Hong Son" or "Maha Sarakham" or Mukdahan or "Nakhon Nayok" or "Nakhon Pathom" or "Nakhon Phanom" or "Nakhon Ratchasima" or "Nakhon Sawan" or "Nakhon Thammarat" or Nan or Narathiwat or "Nong Bua Lamphu" or "Nong Khai" or Nonthaburi or "Pathum Thani" or Pattani or "Phang Nga" or Phatthalung or Phayao or Phetchabun or Phetchaburi or Phichit or Phitsanulok or "Phra Nakhon Si Ayutthaya" or Phrae or Phuket or Prachinburi or "Prachuap Khiri Khan" or Ranong or Ratchaburi or Rayong or "Roi Et" or "Sa Kaeo" or "Sakon Nakhon" or "Samut Prakan" or "Samut Sakjon" or "Samut Songkhram" or Saraburi or Satun or "Sing Buri" or Sisaket or Songkhla or Sukhothai or "Suphan Buri" or "Surat Thani" or Surin or Tak or Trang or Trat or "Ubon Ratchathani" or "Udon Thani" or "Uthai Thani" or Uttaradit or Yala or Yasothon)
18	S10 OR S11 OR S12 OR S13 OR S14 OR S15 OR S16 OR S17
19	S9 AND S18
20	("randomi?ed controlled trial" or "controlled trial" or "control trial" or "controlled study" or "control group?" or "experimental study" or "non-randomi?ed trial" or "pilot trial" or randomi?e? or trial or randomly)
21	(cohort or "controlled before-after study" or "before-after study" or "pre-post evaluation*" or "endline evaluation*" or endline or "difference-in-differences" or pretest or posttest)
22	S20 OR S21
23	S19 AND S22
24	S19 AND S22 limits (2015-Current)

PROQUEST Dissertation and Theses

#	Search term
1	noft(menstru* or menses or catamenia or menarche or dysmenor* or endometrios?s or amenor*ea or menor*agi* or oligomenor* or "premenstrual syndrome")
2	noft(menst* NEAR/3 (period or cycle or disorder* or pain*))
3	noft(period? NEAR/1 (pain* or disorder* or irregular* or infrequent or abnormal))
4	noft("heavy period?" or "light period*" or "period discomfort*")
5	S1 OR S2 OR S3 OR S4
6	noft(Pacific* or Melanesia* or Micronesia* or Polynesia* or "Southeast* Asia" or "South East Asia" or "Indochin* Peninsula" or (Fiji* or Ba or Bua or Cakaudrove or Kadavu or Lau or Lomaiviti or Macuata or "Nadroga Navosa" or Naitasiri or Namosi or Ra or Rewa or Serua or Tailevu or Rotuma) or (Yap or Chuuk or Pohnpei or Kosrae) or (Kiribati or "Gilbert Islands" or "Phoenix Islands" or "Line Islands" or Gilbertese))
7	noft("Papua New Guinea*" or Papua Niugini* or "Papua Niu Gini*" or (Samoa* or Savaii or Upolu or "Navigator island*" or Tuamasaga or Aana or "Aiga i le Tai" or Atua or "Vaa o Fonoti" or Faasaleleaga or Gagaemauga or Gagaifomauga or Vaisigano or Satupaitea or Palauli or Afega or Leulumoega or Mulifanua or Lufilufi or Samamea or Safotulafai or Saleaula or Aopo or Asau or Satupaitea or Vailoa or Apia or Salelologa) or ("Solomon Island*" or "Central Province" or Choiseul or Guadalcanal or Isabel or "Makira Ulawa" or Malaita or "Rennell and Bellona" or Temotu or "Capital Territory") or (Vanuatu* or "New Hebride*" or Malampa or Penama or Sanma or Shefa or Tafea or Torba))
8	noft(Cambodia* or Kampuchea* or "khmer republic" or "Banteay Meanchey" or Battambang or "Kampong Cham" or "Kampong Chhnang" or "Kampong Speu" or "Kampong Thom" or Kampot or Kandal or "Koh Kong" or Kratie or Mondulkiri or "Phnom Penh" or "Preah Vihear" or Prey or Veng or Pursat or Ratanakiri or "Siem Reap" or "Preah Sihanouk" or "Stung Treng" or "Svay Rieng" or Takeo or Oddar or Meanchey or Kep or Pailin or "Tboung Khmum" or (Indonesia* or Celebes or "East Indies" or Aceh or "Bangka Belitung Island*" or Bengkulu or Jambi or Lampung or Sumatra* or Riau or Banten or Java* or Jakarta* or Yogyakarta* or Kalimantan* or Bali* or "Nusa Tenggara*" or Sulawesi* or Gorontalo* or Maluku* or Papua or "West New Guinea" or "Irian Jaya" or Madoera or Madura or Malay* or West Irian))
9	noft(Laos or laotian or "Attapeu*" or "Bokeo*" or "Bolikhamxai*" or "Champasak*" or "Houaphanh*" or Khammouane or "Luang Namtha*" or "Luang Prabang*" or "Oudomxay*" or "Phongsaly*" or "Salavan*" or "Savannakhet*" or "Vientiane*" or "Sainyabuli*" or "Sekong*" or "Xaisomboun*" or "Xiangkhouang*" or (Mongolia* or Arkhangai or Bayankhongor or "Bayan Olgii" or "Darkhan Uul Dornod" or Dornogovi or Dundgovi or "Govi Altai" or Khentii or Govisumber or Khovd or Khovsgol or Moron or Orkhon or mnogovi or Ovorkhangai or Selenge or Sukhbaatar or Tov or Uvs or Zavkhan or Ulaanbaatar) or (Burma or burmese or myanma or Myanmar or Yangon or Mandalay* or Magway* or Sagaing or Bago or Ayeyarwady or Tanintharyi or Kachin or Kayah or Kayin or Chin or Mon or Rakhine or Shan))
10	noft(Philippine* or filipin* or "National Capital Region" or "Cordillera Administrative" or Ilocos or Cagayan or Calabarzon or "Southwestern Tagalog" or Bicol or Western Visayas or "Central Visayas" or "Eastern Visayas" or Zamboanga or "Northern Mindanao" or Davao or Soccsksargen or Caraga or Bangsamoro or Cavite or Bulacan or Laguna or Rizal or Cebu or Pangasinan or Batangas or "Negros Occidental" or Pampanga or "Nueva Ecija" or Iloilo or Quezon or "Camarines Sur" or Isabela or Leyte or Bukidnon or Tarlac or Cotabato or "Negros Oriental" or Bohol or Albay or Maguindanao or Cagayan or "Lanao del Sur" or "Davao del Norte" or "Zamboanga del Sur" or "Zamboanga del Norte" or Sulu or "South Cotabato" or "Misamis Oriental" or Palawan or Masbate or "Oriental Mindoro" or "Sultan Kudarat" or Bataan or Sorsogon or "La Union" or Capiz or "Cotabato City" or Butuan or Koronadal or "Davao City" or "Cagayan de Oro" or Pagadian or Tacloban or Iloilo or Legazpi or Calapan or Calamba or "San Fernando" or Tuguegarao or Baguio or Manila or "Quezon City" or Caloocan or "Zamboanga City" or "Cebu City" or Taguig or Pasig or Valenzuela or "General Santos" or Paranaque or (Timor* or

	Bobonaro or Liquica or Dili or Baucau or Manatuto or Lautem or “Cova Lima” or Ainaro or Manufahi or Viqueque or Ermera or Aileu or “Oecussi Ambeno”))
11	noft(Vietnam* or “Viet Nam*” or “Dong Bac Bo” or “Tay Bac Bo” or “Red River Delta” or “Dong Bang Song Hong” or “North Central Coast” or “Ha Noi Kinh” or “South Central Coast” or “Bac Trung Bo” or “Central Highlands” or “Duyen Hai Nam Trung Bo” or “Tay Nguyen” or “Southeast” or “Dong Nam Bo” or “Mien Dong” or “Mekong River Delta” or “Dong Bang Song Cuu Long” or “Tay Nam Bo Mien Tay” or “Bac Giang” or “Phu Tho” or “Quang Ninh” or “Thai Nguyen” or “Ha Tay” or “Hai Duoong” or “Hung Yen” or “Nam Dinh” or “Thai Binh” or “Vinh Phuc” or “Ha No” or “Hai Phong” or “Ha Tinh” or “Nghe An” or “Thanh Hoa” or “Thua Thien Hue” or “Binh dinh” or “Khanh Hoa” or “Quang Nam” or “Quang Ngai” or “Binh Thuan” or “Dak Lak” or “Gia Lai” or “Lam Dong” or “Dong Nai” or “Ho Chi Minh City” or “An Giang” or “Ben Tre” or “Ca Mau” or “Dong Thap” or “Kien Giang” or “Long An” or “Soc Trang” or “Tien Giang” or “Vinh Long” or “Can Tho” or “Viet Tri” or “Ha Long” or “Thai Nguyen” or “Ha Dong” or “Hai Duong” or “Hung Yen” or “Nam Dinh” or “Thai Binh” or “Vinh Yen” or “Ha Tinh” or Vinh or “Thanh Hoa” or Hue or “Qui Nhon” or “Nha Trang” or “Tam Ky” or “Quang Ngai” or “Phan Thiet” or “Buon Ma Thuot” or Pleiku or “Da Lat” or “Bien Hoa” or “Long Xuyen” or “Ben Tre” or “Ca Mau” or “Cao Lanh” or “Rach Gia” or “Tan An” or “Soc Trang” or “My Tho” or “Tra Vinh” or “Vinh Long”)
12	noft(China or Chinese or Zhongguo or “Zhong gou*” or “Hong Kong” or Macau or Beijing or Tianjin or Hebei or Liaoning or Shanghai or Jiangsu or Zhejiang or Fujian or Shandong or Guangdong or Hainan or Shanxi or Jilin or Heilongjiang or Anhui or Jiangxi or Henan or Hubei or Hunan or Inner Mongolia or Guangxi or Chongqing or Sichuan or Guizhou or Yunnan or Tibet or Shaanxi or Gansu or Qinghai or Ningxia or Xinjiang or (Korea* or Chagang or Hamgyong or Hwanghae or Kangwon or Pyongan or Ryanggang) or (malay* or “Kuala Lumpur” or Johor or Kedah or Kelantan or Malacca or “Negeri Sembilan” or Pahang or Penang or Perak or Perlis or Sabah or Sarawak or Selangor or Terengganu))
13	noft(Thai* or Siam or Bangkok or “Amnat Charoen” or “Ang Thong” or “Bueng Kan” or Buriram or Chachoengasao or “Chai Nat” or Chaiyaphum or Chanthaburi or Chiang Mai or “Chiang Mai” or “Chiang Rai” or Chonburi or Chumphon or Kalasin or “Kamphaeng Phet” or Kanchanaburi or “Khon Kaen” or Krabi or Lampang or Lamphun or Loei or Lopburi or “Mae Hong Son” or “Maha Sarakham” or Mukdahan or “Nakhon Nayok” or “Nakhon Pathom” or “Nakhon Phanom” or “Nakhon Ratchasima” or “Nakhon Sawan” or “Nakhon Thammarat” or Nan or Narathiwat or “Nong Bua Lamphu” or “Nong Khai” or Nonthaburi or “Pathum Thani” or Pattani or “Phang Nga” or Phatthalung or Phayao or Phetchabun or Phetchaburi or Phichit or Phitsanulok or “Phra Nakhon Si Ayutthaya” or Phrae or Phuket or Prachinburi or “Prachuap Khiri Khan” or Ranong or Ratchaburi or Rayong or “Roi Et” or “Sa Kaeo” or “Sakon Nakhon” or “Samut Prakan” or “Samut Sakjon” or “Samut Songkhram” or Saraburi or Satun or “Sing Buri” or Sisaket or Songkhla or Sukhothai or “Suphan Buri” or “Surat Thani” or Surin or Tak or Trang or Trat or “Ubon Ratchathani” or “Udon Thani” or “Uthai Thani” or Uttaradit or Yala or Yasothon)
14	S6 OR S7 OR S8 OR S9 OR S10 OR S11 OR S12 OR S13
15	S5 AND S14
16	(“randomi?ed controlled trial” or “controlled trial” or “control trial” or “controlled study” or “control group?” or “experimental study” or “non-randomi?ed trial” or “pilot trial” or randomi?e? or trial or randomly)
17	(cohort or “controlled before-after study” or "before-after study" or “pre-post evaluation*” or “endline evaluation*” or endline or “difference-in-differences” or pretest or posttest)
18	S16 OR S17
24	S15 AND S18
25	S15 AND S18 limits (2015-Current)

CINAHL

	**Search term**
1	Menstruation/
2	(menstru* or menses or catamenia or menarche or dysmenor* or endometrios?s or amenor*ea or menor*agi* or oligomenor* or “premenstrual syndrome”).ti,ab,kw
3	(menst* N3 (period or cycle or disorder* or pain*)).ti,ab,kw
4	(period? N1 (pain* or disorder* or irregular* or infrequent or abnormal)).ti,ab,kw
5	(“heavy period?” or “light period*” or “period discomfort*”).ti,ab,kw
6	1 or 2 or 3 or 4 or 5
7	(Pacific* or Melanesia* or Micronesia* or Polynesia* or “Southeast* Asia” or “South East Asia” or “Indochin* Peninsula” or (Fiji* or Ba or Bua or Cakaudrove or Kadavu or Lau or Lomaiviti or Macuata or “Nadroga Navosa” or Naitasiri or Namosi or Ra or Rewa or Serua or Tailevu or Rotuma) or (Yap or Chuuk or Pohnpei or Kosrae) or (Kiribati or “Gilbert Islands” or “Phoenix Islands” or “Line Islands” or Gilbertese)).ti,ab,kw
8	(“Papua New Guinea*” or Papua Niugini*” or “Papua Niu Gini*” or (Samoa* or Savaii or Upolu or “Navigator island*” or Tuamasaga or Aana or “Aiga i le Tai” or Atua or "Vaa o Fonoti" or Faasaleleaga or Gagaemauga or Gagaifomauga or Vaisigano or Satupaitea or Palauli or Afega or Leulumoega or Mulifanua or Lufilufi or Samamea or Safotulafai or Saleaula or Aopo or Asau or Satupaitea or Vailoa or Apia or Salelologa) or (“Solomon Island*” or “Central Province” or Choiseul or Guadalcanal or Isabel or “Makira Ulawa” or Malaita or "Rennell and Bellona" or Temotu or “Capital Territory”) or (Vanuatu* or "New Hebride*" or Malampa or Penama or Sanma or Shefa or Tafea or Torba)).ti,ab,kw
9	(Cambodia* or Kampuchea* or “khmer republic” or “Banteay Meanchey” or Battambang or “Kampong Cham” or “Kampong Chhnang” or “Kampong Speu” or “Kampong Thom” or Kampot or Kandal or “Koh Kong” or Kratie or Mondulkiri or “Phnom Penh” or “Preah Vihear” or Prey or Veng or Pursat or Ratanakiri or “Siem Reap” or “Preah Sihanouk” or “Stung Treng” or “Svay Rieng” or Takeo or Oddar or Meanchey or Kep or Pailin or “Tboung Khmum” or (Indonesia* or Celebes or “East Indies” or Aceh or “Bangka Belitung Island*” or Bengkulu or Jambi or Lampung or Sumatra* or Riau or Banten or Java* or Jakarta* or Yogyakarta* or Kalimantan* or Bali* or “Nusa Tenggara*” or Sulawesi* or Gorontalo* or Maluku* or Papua or “West New Guinea” or “Irian Jaya” or Madoera or Madura or Malay* or West Irian)).ti,ab,kw

10	(Laos or laotian or “Attapeu*” or “Bokeo*” or “Bolikhamxai*” or “Champasak*” or “Houaphanh*” or Khammouane or “Luang Namtha*” or “Luang Prabang*” or “Oudomxay*” or “Phongsaly*” or “Salavan*” or “Savannakhet*” or “Vientiane*” or “Sainyabuli*” or “Sekong*” or “Xaisomboun*” or “Xiangkhouang*” or (Mongolia* or Arkhangai or Bayankhongor or “Bayan Olgii” or “Darkhan Uul Dornod” or Dornogovi or Dundgovi or “Govi Altai” or Khentii or Govisumber or Khovd or Khovsgol or Moron or Orkhon or mnogovi or Ovorkhangai or Selenge or Sukhbaatar or Tov or Uvs or Zavkhan or Ulaanbaatar) or (Burma or burmese or myanma or Myanmar or Yangon or Mandalay* or Magway* or Sagaing or Bago or Ayeyarwady or Tanintharyi or Kachin or Kayah or Kayin or Chin or Mon or Rakhine or Shan)).ti,ab,kw
11	(Philippine* or filipin* or “National Capital Region” or “Cordillera Administrative” or Ilocos or Cagayan or Calabarzon or “Southwestern Tagalog” or Bicol or Western Visayas or “Central Visayas” or “Eastern Visayas” or Zamboanga or “Northern Mindanao” or Davao or Soccsksargen or Caraga or Bangsamoro or Cavite or Bulacan or Laguna or Rizal or Cebu or Pangasinan or Batangas or “Negros Occidental” or Pampanga or “Nueva Ecija” or Iloilo or Quezon or “Camarines Sur” or Isabela or Leyte or Bukidnon or Tarlac or Cotabato or “Negros Oriental” or Bohol or Albay or Maguindanao or Cagayan or "Lanao del Sur" or "Davao del Norte" or "Zamboanga del Sur" or "Zamboanga del Norte" or Sulu or “South Cotabato” or “Misamis Oriental” or Palawan or Masbate or “Oriental Mindoro” or “Sultan Kudarat” or Bataan or Sorsogon or "La Union" or Capiz or “Cotabato City” or Butuan or Koronadal or “Davao City” or "Cagayan de Oro" or Pagadian or Tacloban or Iloilo or Legazpi or Calapan or Calamba or "San Fernando" or Tuguegarao or Baguio or Manila or “Quezon City” or Caloocan or “Zamboanga City” or “Cebu City” or Taguig or Pasig or Valenzuela or “General Santos” or Paranaque or (Timor* or Bobonaro or Liquica or Dili or Baucau or Manatuto or Lautem or “Cova Lima” or Ainaro or Manufahi or Viqueque or Ermera or Aileu or “Oecussi Ambeno”)).ti,ab,kw
12	(Vietnam* or “Viet Nam*” or “Dong Bac Bo” or “Tay Bac Bo” or “Red River Delta” or “Dong Bang Song Hong” or “North Central Coast” or “Ha Noi Kinh” or “South Central Coast” or “Bac Trung Bo” or “Central Highlands” or “Duyen Hai Nam Trung Bo” or “Tay Nguyen” or “Southeast” or “Dong Nam Bo” or “Mien Dong” or “Mekong River Delta” or “Dong Bang Song Cuu Long” or “Tay Nam Bo Mien Tay” or “Bac Giang” or “Phu Tho” or “Quang Ninh” or “Thai Nguyen” or “Ha Tay” or “Hai Duoong” or “Hung Yen” or “Nam Dinh” or “Thai Binh” or “Vinh Phuc” or “Ha No” or “Hai Phong” or “Ha Tinh” or “Nghe An” or “Thanh Hoa” or “Thua Thien Hue” or “Binh dinh” or “Khanh Hoa” or “Quang Nam” or “Quang Ngai” or “Binh Thuan” or “Dak Lak” or “Gia Lai” or “Lam Dong” or “Dong Nai” or “Ho Chi Minh City” or “An Giang” or “Ben Tre” or “Ca Mau” or “Dong Thap” or “Kien Giang” or “Long An” or “Soc Trang” or “Tien Giang” or “Vinh Long” or “Can Tho” or “Viet Tri” or “Ha Long” or “Thai Nguyen” or “Ha Dong” or “Hai Duong” or “Hung Yen” or “Nam Dinh” or “Thai Binh” or “Vinh Yen” or “Ha Tinh” or Vinh or “Thanh Hoa” or Hue or “Qui Nhon” or “Nha Trang” or “Tam Ky” or “Quang Ngai” or “Phan Thiet” or “Buon Ma Thuot” or Pleiku or “Da Lat” or “Bien Hoa” or “Long Xuyen” or “Ben Tre” or “Ca Mau” or “Cao Lanh” or “Rach Gia” or “Tan An” or “Soc Trang” or “My Tho” or “Tra Vinh” or “Vinh Long”).ti,ab,kw
13	(China or Chinese or Zhongguo or “Zhong gou*” or “Hong Kong” or Macau or Beijing or Tianjin or Hebei or Liaoning or Shanghai or Jiangsu or Zhejiang or Fujian or Shandong or Guangdong or Hainan or Shanxi or Jilin or Heilongjiang or Anhui or Jiangxi or Henan or Hubei or Hunan or Inner Mongolia or Guangxi or Chongqing or Sichuan or Guizhou or Yunnan or Tibet or Shaanxi or Gansu or Qinghai or Ningxia or Xinjiang or (Korea* or Chagang or Hamgyong or Hwanghae or Kangwon or Pyongan or Ryanggang) or (malay* or “Kuala Lumpur” or Johor or Kedah or Kelantan or Malacca or “Negeri Sembilan” or Pahang or Penang or Perak or Perlis or Sabah or Sarawak or Selangor or Terengganu)).ti,ab,kw
14	(Thai* or Siam or Bangkok or “Amnat Charoen” or “Ang Thong” or “Bueng Kan” or Buriram or Chachoengasao or “Chai Nat” or Chaiyaphum or Chanthaburi or Chiang Mai or “Chiang Mai” or “Chiang Rai” or Chonburi or Chumphon or Kalasin or “Kamphaeng Phet” or Kanchanaburi or “Khon Kaen” or Krabi or Lampang or Lamphun or Loei or Lopburi or “Mae Hong Son” or “Maha Sarakham” or Mukdahan or “Nakhon Nayok” or “Nakhon Pathom” or “Nakhon Phanom” or “Nakhon Ratchasima” or “Nakhon Sawan” or “Nakhon Thammarat” or Nan or Narathiwat or “Nong Bua Lamphu” or “Nong Khai” or Nonthaburi or “Pathum Thani” or Pattani or “Phang Nga” or Phatthalung or Phayao or Phetchabun or Phetchaburi or Phichit or Phitsanulok or “Phra Nakhon Si Ayutthaya” or Phrae or Phuket or Prachinburi or “Prachuap Khiri Khan” or Ranong or Ratchaburi or Rayong or “Roi Et” or “Sa Kaeo” or “Sakon Nakhon” or “Samut Prakan” or “Samut Sakjon” or “Samut Songkhram” or Saraburi or Satun or “Sing Buri” or Sisaket or Songkhla or Sukhothai or “Suphan Buri” or “Surat Thani” or Surin or Tak or Trang or Trat or “Ubon Ratchathani” or “Udon Thani” or “Uthai Thani” or Uttaradit or Yala or Yasothon).ti,ab,kw
15	7 or 8 or 9 or 10 or 11 or 12 or 13 or 14
16	6 and 15
17	(MH "Clinical Trials") OR (MH "Randomized Controlled Trials")
18	(MH "Controlled Before-After Studies")
19	(MH "Pilot Studies") OR (MH "Quantitative Studies")
20	(MH "Pretest-Posttest Design") OR (MH "Pretest-Posttest Control Group Design")
21	(“randomi?ed controlled trial” or “controlled trial” or “control trial” or “controlled study” or “control group?” or “experimental study” or “non-randomi?ed trial” or “pilot trial” or randomi?e? or trial or randomly).mp.
22	(cohort or “controlled before-after study” or "before-after study" or “pre-post evaluation*” or “endline evaluation*” or endline or “difference-in-differences” or pretest or posttest).mp.
23	(Evaluation OR comparison).mp.
24	(Program* OR policy OR intervention).mp.
25	17 or 18 or 19 or 20 or 21 or 22
26	16 and 23
27	limit 24 to yr="2015 -Current"

Supplementary Materials 3: Full text screening, excluded studies

Akter M, Khatun S, Biswas HB, Kim HS. Knowledge of menstruation and the practice of hygiene among adolescent girls in Bangladesh. East African Scholars Journal of Medical Sciences. 2019;2(10):605–12.

Reason for exclusion: Ineligible intervention

Al Kiyumi MH. The effects of psychoeducation based on the cognitive-behavioral approach on premenstrual syndrome symptoms: a randomized controlled trial. Perspectives in psychiatric care [Internet]. 2021;57(1):408. Available from: http://ovidsp.ovid.com/ovidweb.cgi?T=JS&PAGE=reference&D=cctr&NEWS=N&AN=CN-02300637

Reason for exclusion: Ineligible participant characteristics

Alor SK, Anaba EA, Adongo PB. Correlates of absenteeism at work, school and social activities during menstruation: Evidence from the 2017/2018 Ghana Multiple Indicator Cluster Survey. PLoS One. 2022;17(6):e0270848.

Reason for exclusion: Ineligible participant characteristics

Buggio L, Barbara G, Facchin F, Frattaruolo MP, Aimi G, Berlanda N. Self-management and psychological-sexological interventions in patients with endometriosis: Strategies, outcomes, and integration into clinical care. International Journal of Women's Health [Internet]. 2017;9:281–93. Available from: http://ovidsp.ovid.com/ovidweb.cgi?T=JS&PAGE=reference&D=emed18&NEWS=N&AN=616505273

Reason for exclusion: Ineligible study design

Chen L, Tang L, Guo S, Kaminga AC, Xu H. Primary dysmenorrhea and self-care strategies among Chinese college girls: A cross-sectional study. BMJ Open [Internet]. 2019;9(9):e026813. Available from: http://ovidsp.ovid.com/ovidweb.cgi?T=JS&PAGE=reference&D=emed20&NEWS=N&AN=629373531

Reason for exclusion: Ineligible intervention

Choi HK, Lee SH, Yang SY. Development of an integrated mobile application for lifestyle modification in women with polycystic ovarian syndrome. Journal of Clinical Nursing [Internet]. 2022;No-Specified. Available from: http://ovidsp.ovid.com/ovidweb.cgi?T=JS&PAGE=reference&D=psyc18&NEWS=N&AN=2022-33478-001

Reason for exclusion: Ineligible study design

Effect of Progressive Muscle Relaxation Exercise on Primary Dysmenorrhea Menstrual Symptoms and Quality of Life. Effect of Progressive Muscle Relaxation Exercise on Primary Dysmenorrhea Menstrual Symptoms and Quality of Life Randomized Controlled Study [Internet]. 2022; Available from: http://ovidsp.ovid.com/ovidweb.cgi?T=JS&PAGE=reference&D=cctr&NEWS=N&AN=CN-02398311

Reason for exclusion: Ineligible participant characteristics

Harrington R, Redman-MacLaren M, Harvey N, Puia M, Carlisle K, Larkins S. Barriers and enablers to using contraceptives for family planning at Atoifi Hospital, East Kwaio, Solomon Islands. Pacific Journal of Reproductive Health. 2020;1:586–97.

Reason for exclusion: Ineligible study design

Heat Application on Depression, Anxiety, Menstrual Attitude and Dysmenorrhea. The Effects of Heat Application on Depression, Anxiety, Menstrual Attitude and Severity of Dysmenorrhea at 4 Menstrual Cycle [Internet]. 2020; Available from: http://ovidsp.ovid.com/ovidweb.cgi?T=JS&PAGE=reference&D=cctr&NEWS=N&AN=CN-02174400

Reason for exclusion: Ineligible participant characteristics

Htun NN, Laosee O, Rattanapan C. Factors that influence menstrual hygiene management in adolescent girls in Mudon Township, Mon State, Myanmar. Journal of Health Science and Medical Research. 2021;39(3):207–17.

Reason for exclusion: Ineligible intervention

Huggett C, Mohamed Y, Macintyre A, McSkimming D, Barrington D, Durrant K, et al. Formative menstrual hygiene management research in the Pacific. In: Water and Health Conference. 2017.

Reason for exclusion: Ineligible intervention

Jay JE, Chan KY. Girls Education Around the World: Exploring Intervention Methods of Nonprofits in Israel, Thailand, Cambodia, and Sri Lanka used to increase Girls Education. 2018;

Reason for exclusion: Ineligible study design

Kazemi M, Pierson RA, McBreairty LE, Chilibeck PD, Zello GA, Chizen DR. A randomized controlled trial of a lifestyle intervention with longitudinal follow-up on ovarian dysmorphology in women with polycystic ovary syndrome. Clinical Endocrinology [Internet]. 2020;92(6):525. Available from: http://ovidsp.ovid.com/ovidweb.cgi?T=JS&PAGE=reference&D=cctr&NEWS=N&AN=CN-02098896

Reason for exclusion: Ineligible intervention

Liu R, Li M, Wang P, Yu M, Wang Z, Zhang GZ. Preventive online and offline health management intervention in polycystic ovary syndrome. World Journal of Clinical Cases [Internet]. 2022;10(10):3060–8. Available from: http://ovidsp.ovid.com/ovidweb.cgi?T=JS&PAGE=reference&D=emexb&NEWS=N&AN=2017791100

Reason for exclusion: Ineligible intervention

Mactaggart I, Baker S, Bambery L, Iakavai J, Kim MJ, Morrison C, et al. Water, women and disability: Using mixed-methods to support inclusive wash programme design in Vanuatu. The Lancet Regional Health-Western Pacific. 2021;8:100109.

Reason for exclusion: Ineligible intervention

Menstrual Health Education's Impact on Knowledge, Attitudes and Self Care Behavior of Dysmenorrheal Adolescents. Impact of Menstrual Health Education on Knowledge, Attitudes and Self Care Behavior of Female Adolescents With Primary Dysmenorrhea [Internet]. 2022; Available from: http://ovidsp.ovid.com/ovidweb.cgi?T=JS&PAGE=reference&D=cctr&NEWS=N&AN=CN-02392202

Reason for exclusion: Ineligible intervention

The effect of menopausal self-care application on the severity of menopausal symptoms in postmenopausal women. Designing a menopausal self-care application and examining its effect on the severity of menopausal symptoms in postmenopausal women referring to health centers in Jiroft in 2020 [Internet]. 2021; Available from: http://ovidsp.ovid.com/ovidweb.cgi?T=JS&PAGE=reference&D=cctr&NEWS=N&AN=CN-02280641

Reason for exclusion: Ineligible participant characteristics

Zhao J, Ketlhoafetse A, Liu X, Cao Y. Comparative effectiveness of aerobic exercise versus Yi Jin Jing

on ovarian function in young overweight/obese women with polycystic ovary syndrome: study protocol for a randomized controlled trial. Trials [Internet]. 2022;23(1):459. Available from: http://ovidsp.ovid.com/ovidweb.cgi?T=JS&PAGE=reference&D=emexb&NEWS=N&AN=2017619452

Reason for exclusion: Ineligible intervention

Supplementary Materials 4: Data extraction form

Study title:

Study details		
Author		
Country		
Location (region, province)		
Setting (urban/rural) (university/school)		
Year of data collection		
Main research topic		
Study type		
Study aims		
Trial registration/pre registration		
Participants		
Number		
Age range		
Socio-demographics (gender, country and local context ie: WASH status, healthcare status, menstrual practices as relevant to the intervention),		
Intervention		
Description		
Duration		
Mode of delivery		
Location of delivery		
Additional interventions delivered		
Participant uptake		
Control		
Description		
Outcome		
Timing of outcome assessment		
Method of outcome assessment		
Outcomes assessed		
Measures used		
Data analysis		
Type of analysis		
Adjustment for baseline differences		
Intention-to-treat analysis		
Data clustering/adjustment for data clustering		
Additional notes		
Funding sources		
Government/NGO affiliation		
Author contact information		

Supplementary Materials 5: Risk of Bias Assessment

Study ID	Outcomes being assessed	Randomisation (RoB 2)			Deviation from the intended intervention (RoB 2 and ROBNIS-I)	Missing data (RoB 2 and ROBNIS-I)	Measurement of outcomes (RoB 2 and ROBNIS-I)	Selection of the reported results (RoB 2 and ROBNIS-I)	Overall
		**Confounding** (ROBINS-I)	**Selection of participants** (ROBNIS-I)	**Classification of interventions** (ROBINS-I)					
**Single MH requirement addressed**									
Al Ajeel 2020[16]	• PD Knowledge<br>• PD Self-care	**High**<br>Schools self-selected condition. Balance tested, however no adjustment for unmeasured school-based characteristics such as supportive environment for MH/SRH.	**Low**<br>Selection of participants into the study was equivalent to the target trial.	**Low**<br>All participants who would have been eligible for the target trial were included in the study.	**Low**<br>Clustered allocation, low risk of contamination.	**Low**<br>All outcomes were presented in tables for total number of participants	**Low – Knowledge**<br>Objective outcome, lack of blinding to the intervention condition could not influence the outcome.<br>**High – Self-care**<br>Subjective, self-reported outcome with no participant blinding.	**Some concerns**<br>No pre-registration or protocol reported. Multiple outcomes reported, consistent with methods.	**Serious (High)**
Bustan 2018	• Pain	**Some concerns**<br>Allocation to groups reported as non-random, no further information.	**Low**<br>Selection of participants into the study was equivalent to the target trial.	**Low**<br>Participants assigned to conditions. All participants who would have been eligible for the target trial were included in the study.	**High**<br>Lack of reporting on adherence and supervision of intervention. Contamination likely among students in the same college.	**Some concerns**<br>Participant numbers and lost-to-follow-up not reported.	**High**<br>Subjective, self-reported outcome. Participants unable to be blinded to intervention condition.	**Some concerns**<br>No pre-registration or stated protocol. Single outcome reported.	**Serious (High)**
Djupri 2022	• Pain	**High**<br>Allocation method not reported, described by authors as "non-equivalent" control. No adjustment in outcome analysis.	**Low**<br>Selection of participants into the study was equivalent to the target trial.	**Low**<br>Participants assigned to conditions. All participants who would have been eligible for the target trial were included in the study.	**Some concerns**<br>Lack of reporting on adherence. Contamination possible among students in the same school, short intervention timeframe mitigates risk.	**Low**<br>All data is available, although the data analysis provided is very minimal	**High**<br>Subjective, self-reported outcome. Participants unable to be blinded to intervention condition.	**Some concerns**<br>No pre-registration or protocol reported. Multiple outcomes reported, consistent with methods.	**Serious (High)**
Downing 2021	• Acceptability of sanitary product	**High**<br>Lack of allocation concealment from researchers. No information on randomization protocol. Based on the limited information provided, not confident true randomization occurred.			**High**<br>Participant blinding not possible.<br>No mechanism or reporting of adherence.	**High**<br>Endline only assessment. Substantial lost to follow up.<br>Varying participant numbers reported.<br>No adjustment for lost-to-follow-up in ITT analysis.	**High**<br>Subjective, self-reported outcome(s) with no participant blinding.	**High**<br>No pre-registration or protocol. Reporting suggests risk of selected outcome reporting.	**Serious (High)**
Juan 2016	• PD Symptoms<br>• Pain<br>• Quality of life	**Some Concerns**<br>Random allocation method not reported, allocation concealment not reported, Baseline balance checks appropriate and suggest balance.			**Some concerns**<br>Blinding educators or participants not possible. Intervention was self-management, Lack of reporting on adherence.	**Low**<br>No missing data reported.	**Some Concerns – Pain and QoL**<br>Appropriate, self-reported and blinding unfeasible.	**Some concerns**<br>No pre-registration or protocol. Multiple outcomes reported consistent with methods.	**Moderate (Some concerns)**
Nguyen 2015	• SRH Knowledge	**Some concerns**<br>Allocation to groups reported as non-random,	**Low**<br>Selection of participants into the study	**Low**<br>All participants who would	**High**<br>Participant blinding not possible.	**Some concerns**<br>Endline only assessment.	**Low**<br>Objective outcome, lack of blinding to the	**Some concerns**<br>No pre-registration	**Serious (High)**

		no further information. No baseline data or comparison.	was equivalent to the target trial.	have been eligible for the target trial were included in the study.	Single education session, likely delivered by investigators. Groups allocated within single schools, high likelihood of contamination.	Missing participants not reported, short follow-up window.	intervention condition could not influence the outcome.	or protocol reported. Multiple outcomes reported, consistent with methods.	
Nik Farid 2018	• SRH Knowledge<br>• SRH Attitudes	**Some Concerns**<br>Random allocation method and allocation concealment are not reported.			**Low**<br>Participant blinding not possible.<br>Single education session with post-test data collected immediately after intervention mitigating likelihood of deviation.	**Low**<br>No missing data	**Low – Knowledge**<br>Objective outcome, lack of blinding to the intervention condition could not influence the outcome.<br>**Some Concerns - Attitudes**<br>Subjective outcome, unblinded participants.	**Some concerns**<br>No pre-registration or protocol reported. Multiple outcomes reported, consistent with methods.	**Moderate (Some concerns)**
Rejeki 2021	• Pain	**Some concerns**<br>The authors have reported that the study was randomized. However, randomization and allocation concealment methods not reported.			**High**<br>Lack of reporting on adherence or supervision. Contamination likely among students in the same school, duration of intervention unclear.	**Low**<br>No missing data reported	**High**<br>Subjective, self-reported outcome. Participants unable to be blinded to intervention condition.	**Some concerns**<br>No pre-registration or protocol reported. Multiple outcomes reported, consistent with methods.	**Serious (High)**
Saarul 2020<br><br>**Pre-post study**	• Emotional state<br>• Choice and use of sanitary pads<br>• MH practices<br>• Quality of teaching<br>• MHH Knowledge	**High**<br>No control group.	**Low**<br>Selection of participants into the study was equivalent to the target trial.	**Low**<br>All participants who would have been eligible for the target trial were included in the study.	**Low**<br>Single education session with post-test data collected immediately after intervention mitigating likelihood of deviation.	**Low**<br>No missing data	**Low – Knowledge**<br>Objective outcome, lack of blinding to the intervention condition could not influence the outcome.<br>**Some concerns – Emotional state, choice of sanitary pads, MHH practices, quality of teaching**<br>Subjective outcomes, unblinded participants.	**Some concerns**<br>No pre-registration or protocol reported. Multiple outcomes reported, consistent with methods.	**Serious (High)**
Setyowati 2019	• Knowledge<br>• Emotional response<br>• Attitudes	**High**<br>It is not clear how intervention or control group was determined.	**Low**<br>Selection of participants into the study was equivalent to the target trial.	**Low**<br>All participants who would have been eligible for the target trial were included in the study.	**High**<br>Participant blinding not possible. Engagement with booklet provided as intervention not reported. Contamination likely among students in a single school.	**Low**<br>No missing data. Follow up time not reported.	**Low – Knowledge**<br>Test not susceptible to self-report, subjective reporting.<br>**Some concerns – emotional response and attitudes**<br>Subjective outcome, unblinded participants.	**Some concerns**<br>No pre-registration or protocol reported. Multiple outcomes reported, consistent with methods.	**Serious (High)**
Silitonga 2021	• SRH Knowledge<br>• SRH Attitudes	**Critical**<br>Allocation into groups based on participant characteristics. No subsequent adjustment.	**Low**<br>Selection of participants into the study was equivalent to the target trial.	**Low**<br>All participants who would have been eligible for the target trial were included in the study.	**High**<br>Participant blinding not possible. Contamination likely due to the proximity of the participants to one another and delivery of	**Some concerns**<br>Follow-up time not reported. Participant numbers not reported.	**Low - Knowledge**<br>Objective outcome, lack of blinding to the intervention condition could not influence the outcome.<br>**High -Attitudes**	**High**<br>No pre-registration or protocol. Reporting suggests risk of selected outcome reporting	**Critical**

					multiple sessions over 2 weeks.		Subjective outcome with no participant blinding.		
Sumarah 2017	• Vaginal hygiene practice<br>• Attitudes<br>• Behaviours	**Some concerns**<br>Allocation to groups reported as non-random, no further information.<br>No baseline characteristics assessment, baseline score adjustment.	**Low**<br>Selection of participants into the study was equivalent to the target trial.	**Low**<br>All participants who would have been eligible for the target trial were included in the study.	**Some concerns**<br>Participant blinding not possible.<br>Intervention described as self-learning module, adherence not reported.	**Low**<br>No missing data reported.	**High**<br>Subjective, self-reported outcome with no participant blinding.	**High**<br>No pre-registration or protocol.<br>Reporting suggests risk of selected outcome reporting.	**Serious (High)**
Van Hung 2019	• SRH Knowledge	**Some Concerns**<br>The authors have reported that the study was randomized. Method of randomization and allocation concealment not reported.			**High**<br>Participant blinding not possible.<br>Contamination likely among students in a single school.	**Low**<br>No missing data reported.	**Low**<br>Objective outcome, lack of blinding to the intervention condition could not influence the outcome.	**Some concerns**<br>No pre-registration or protocol reported.<br>Multiple outcomes reported, consistent with methods.	**Serious (High)**
Weerawatsop on 2020	• Satisfaction of menstrual cup<br>• Acceptability of menstrual cup	**Some concerns**<br>Method of randomization and allocation concealment not reported.			**Some concerns**<br>Participant blinding not possible.<br>Self-administered intervention, lack of reporting on adherence.	**Low**<br>Lost-to-follow-up reported (n=1). No missing data.	**Low**<br>The outcome evaluator was blinded from the methods.	**Low**<br>A per-protocol analysis was performed, and all available data was reported.	**Moderate (Some concerns)**
**Multiple MH requirements addressed**									
Grant 2020	• School absenteeism due to menstruation<br>• MHM Knowledge and Attitudes<br>• Supportive social environment	**High**<br>No control group.	**Low**<br>Selection of participants into the study was equivalent to the target trial.	**Low**<br>All participants who would have been eligible for the target trial were included in the study.	**Low**<br>Single education session over 1-day mitigating likelihood of deviation.	**Some concerns**<br>Lost-to-follow-up participants not reported, numbers not reported in tables.	**Low - Knowledge**<br>Objective outcome, lack of blinding to the intervention condition could not influence the outcome.<br>**Some Concerns – Attitudes, student absenteeism and supportive environment**<br>Subjective outcomes, unblinded participants	**Some concerns**<br>No pre-registration or protocol reported.<br>Multiple outcomes reported, consistent with methods.	**Serious (High)**
Greaves 2019	• Missing sport due to period	**High**<br>No control group.<br>Confounding inherently not controllable.	**Low**<br>Selection of participants into the study was equivalent to the target trial.	**Low**<br>All participants who would have been eligible for the target trial were included in the study.	**High**<br>Attendance at education sessions not reported.<br>Participants self-reported use of provided product, lack of reporting on adherence.	**High**<br>Participant numbers not reported in tables.<br>Significant lost-to-follow-up. No adjustment in analyses.	**High**<br>Subjective, self-reported outcome(s) with no participant blinding.<br><br>Serious likelihood of bias, NGO provision of complimentary menstrual products to participants.	**Some concerns**<br>No pre-registration or protocol reported.<br>Multiple outcomes consistent with methods.	**Serious (High)**
Su. 2016	• MHM Knowledge<br>• MHM Attitude<br>• Menstrual Confidence	**High**<br>It is not clear how intervention or control group was determined.<br>Inappropriate outcome analysis,	**Low**<br>Selection of participants into the study was equivalent to the target trial.	**Low**<br>All participants who would have been eligible for the target trial were included in the study.	**Low**<br>Participant blinding not possible.<br>Multiple education sessions delivered over five weeks, delivered by the	**Some concerns**<br>Participant numbers not reported.<br>No adjustment for loss to-follow-up	**Low – Knowledge**<br>Test not susceptible to self-report, subjective reporting.<br>**High – Attitudes, confidence and behaviour**	**Some concerns**<br>No pre-registration or protocol reported.<br>Multiple outcomes reported,	**Serious (High)**

	• MH Behaviour	changes in intervention and control groups compared independently. Lack of adjustment for baseline scores.			first author, expected high fidelity.	through ITT analysis.	There was non-random assignment to intervention and control with self-reporting, likely bias of the non-blinded intervention.	consistent with methods	
UnTold Research 2021	• Puberty<br>• Menstruation knowledge and perceptions<br>• MHM	**High**<br>No study design is presented. Allocation not reported. Unclear definition of controls, no confounding control.	**No information.**	**Critical**<br>Intervention status is not well defined. Insufficiently described.	**No information**<br>Unclear how intervention and control allocated or defined, proportion of interventions received by each school unclear.	**High**<br>Reported participant numbers vary by outcome, substantial loss-to follow-up. No adjustment.	**Low – Knowledge**<br>Objective outcome, lack of blinding to the intervention condition could not influence the outcome.<br>**High – MH practices, product awareness, attitudes**<br>Subjective outcome with no participant blinding.	**High**<br>No preregistration or protocol. Outcomes poorly defined in methods. Risk of selective reporting from multiple analyses and outcomes.	**Critical**

Supplementary Materials. 6. Summary of study findings

Note. Studies are grouped into tables by the requirement for menstrual health addressed. Within tables studies are grouped first, by the type of intervention (e.g., education focused on menstrual health only, vs broader SRH education), second, by the outcomes measured in the study. This shows the extent to which there are comparable outcomes within comparable interventions. Studies were either insufficiently comparable, or provided inadequate information to meta-analyse findings (e.g., providing group mean but no standard deviation). Review authors have summarised the study finding for each outcome using data provided in the study. Where feasible we have computed a single improvement score (e.g., the difference in change between the intervention and control), with few studies providing a final effect size or standardised effect estimate.

Information and education interventions

Study ID	Country	Timeframe	Study type	Participants	Intervention and control	Time to follow up	Outcomes	Study Findings	Risk of Bias
Saruul 2022	Mongolia	2020	Pre-post	**Total:** 86 (71 post-menarche) Adolescent 8th grade girls. 2 x schools. Rural setting.	**Intervention:** MH education<br><br>**No control**	Immediate	**MHH Knowledge** (quiz scored as % correct)	Quiz performance improved from 50.7% (SD 25.2) pre-intervention to 82.1% (SD 15.9) post-intervention; an improvement of **31.5 percentage points.**	
Su 2016	China	Not reported	CBA	**Total:** 128 (116 final sample) (Intervention: 66, Control: 62). Post-menarchal adolescent girls. 2 x schools. Urban setting. Aged 12-15 ($\bar{x}$=12.26)	**Intervention:** Interactive MH education<br><br>**Control:** Wait-list control.	Immediate	**Menstrual Knowledge** (scored from 0 to 13)	Mean knowledge score in the intervention group significantly increased by 4.49 while the control group only increased 0.27.<br>Transposed to a %, the intervention group score improvement was **32.4 percentage points** greater than the control.	
							**Menstrual Attitude** (measured using Menstrual Attitudes Questionnaire)	Study authors scored attitudes across subscales including debilitating, bothersome or natural. There were large differences between the intervention and control groups at baseline. Endline comparisons are presented between groups, but do not account for baseline differences, or are presented within-groups (pre to post). Significant within-group improvements were reported for attitudes describing menstruation as debilitating [pre: 2.81 (SD 1.07) post: 3.00 (SD 1.09)], bothersome [pre 2.70 (SD 1.10) post: 2.99 (SD 1.08)], natural and something that can be anticipated. However, within-group comparison for the control also observed improvements in attitudes describing menstruation as bothersome [pre: 2.52 (SD 1.20) post: 2.73 (SD 1.15)].	
Grant 2020	Laos	2017-2019	Pre-post	10 Schools<br>**Total:** 292 (Midterm: 291, Endline: 286) Adolescent school students. Aged 10-19. Rural setting.	**Intervention:** MH Education Session (+ broader WASH)<br><br>**No control**	Immediately after workshop.<br><br>Midline.<br><br>Endline conducted end of 2019	**MHH Knowledge and Attitudes** (combined as a single outcome)	*"Pre- and post-test comparison showed 94% of participants improved knowledge and attitudes of MHM with an average improvement rate of 56% on a 6-point scale. The average rate of improvement was 2.6 points, or almost 50%."*<br>No further data provided.	
							**Seeking social support**	The proportion of participants who reported talking to a teacher before their first period increased from 1% to 49% although the time to menarche in the sample was unclear. The proportion of respondents reporting they would talk to someone about menstruation before their first period increased from 36% to 49% and the proportion reporting they would talk to a teacher increased from 0% to 57%.	
							**School absenteeism due to menstruation**	Study authors state: *"This indicator provides no meaningful data. The percentage was already extremely low at baseline."*	

Sumarah 2017	Indonesia	2016	CBA	**Total:** 80<br>(Intervention: 40, Control: 40)<br>Adolescent girls from two schools.<br>Majority Urban.	**Intervention:** Online vaginal hygiene education<br><br>**Control:** No intervention.	6-months post intervention	**Vaginal Hygiene Behaviour**<br>(Composite index)	Study authors reported a composite hygiene behavioural index improved in the intervention group.<br>Review authors note that included behaviours do not align with UNICEF's menstrual hygiene guidance, and so were not included here. Index included items such as: such as changing panty liners >4x per day, using menstrual pads, using powder to reduce itching, changing underwear twice per day, wearing lose-fitting outfits during menstruation)	
							**Vaginal Hygiene Attitudes**<br>Review authors were unable to determine how attitudes were measured.	Attitudes improved in the control group by a mean of 1.82 (SD5.41), and by a significantly greater 9.28 (SD 5.53) in the intervention group.	
UnTold Research (UNICEF) 2021	Indonesia	2019-2020	CBA	25 schools:<br>(Intervention: 20 Control: 5)<br>Student sample: (Baseline: 911, endline 762). Urban setting.	**Intervention:** MH education + storybook<br><br>**Control:** No control group	Immediate	**Menstrual Knowledge**<br>(rated as 'mid to high' or 'none to low', cut off is unclear)	The proportion of students with mid to high knowledge increased from 56 to 65% in the intervention group, and 66% to 68% in the control, a difference in change of **7 percentage points**. *Dropout was significant and it appears missing data were not imputed. No null-hypothesis significance tests were undertaken.	
							**Menstrual Knowledge and Perceptions**	Review authors were unable to determine whether attitudes (perceptions) were included in the composite knowledge score.	
							**Comfort discussing menstruation**	Girls exposed to intervention programming showed significant (5%) higher agreement (58%, compared to a baseline of 53%) that they have a person they can trust to ask questions about their body, compared to girls attending control schools who showed no significant increase in agreement (2%) (51%, compared to a baseline of 53%).<br>Intervention group increased self-reported comfort discussing menstruation with the following individuals, beyond the change reported in the control group:<br>Sister: 0% | Mother: -8% | Female friends: 3%<br>Female teachers: 6% | Health Officer: 5%<br>No statistical significance tests were undertaken.	
Nik Farid 2018	Malaysia	2013-2015	RCT	**Total:** 209<br>(Intervention: 101, Control: 108)<br>Adolescent school students. Aged 12. Urban setting.	**Intervention:** Online SRH education<br><br>**Comparison:** In-person SRH education	Immediate	**SRH Knowledge**<br>(mean score on a test, score range unclear)	Both modes of intervention, in person and online, showed an improvement. Knowledge increased in the online group by a mean 3.88, and by 4.29 in the in-person group.<br>Review authors note that knowledge questions included items that do not align with Comprehensive Sexuality Education Guidance, e.g., "How to avoid masturbating", or that may not reflect knowledge e.g., "what are the factors that influence sexual feelings" and "How do I avoid pornography".	
Silitonga 2021	Indonesia	Not reported	CBA	**Total:** 24 (Grp A:8, Grp B:8, Grp C:8)<br>Female migrant workers. Age reported as: Adults (63% <30, 29% 30-35, 8% >35). Urban setting.	**Intervention 1 & 2:** SRH education with varying case studies<br><br>**Comparison/Control**: SRH education no case studies	Not reported	**SRH Knowledge and Attitudes combined** (70% Knowledge, 30% Attitude measured together)	Intervention group 1 showed an increase in SRH knowledge and attitudes of 7.38 percentage points after the intervention, while intervention group 2 showed 7.75 percentage point increase. The control group receiving education with no case studies also increased by 4.88 percentage points.<br><br>Statistical comparisons between groups are not clearly reported.	

Setyowati 2019	Indonesia	2018	CBA	**Total:** 174 (Intervention: 87, control: 87). Premenarchal adolescent girls from Madrasah - Islamic school. Aged 9-12. Acehnese. Rural setting.	**SRH education** **Intervention:** Researchers provided a SRH booklet **Control:** No intervention (waitlist). Booklet provided after study completion	Not reported	**Menstruation & Puberty Knowledge** (proportion of participants scoring >7 on a 14 question quiz)	The proportion of participants in the intervention group assessed to have 'good' knowledge was **32.2 percentage points** higher than the control group after the intervention. Study authors did not report pre-test control group knowledge scores, so it is unclear if the repeated test scores contributed to the increased score.	
							**Emotional response**	The proportion of participants in the intervention group who reported 'positive' emotional responses to menstruation was **50.6 percentage points greater** than control group post-intervention (no control-group pre-test reported).	
							**Attitudes towards menstruation**	The proportion of participants in the intervention group reporting positive attitudes to menstruation was **54.1 percentage points greater** than control group (no pre-intervention control scores were reported).	
Van Hung 2019	Vietnam	Not reported	RCT	**Total**: 400 (Intervention: 200, Control: 200) Male and female ethnic-minority students. Age not reported. Rural setting.	**Intervention:** Reproductive health education session **Control:** No intervention	6 months post-intervention	**Knowledge:** timing of menarche (proportion answering single question correctly)	The study reports each knowledge question individually We report the two items most relevant to menstruation. The intervention group had a significantly higher proportion of students answering correctly than the control (93.5 vs 65.5), a difference of **28 percentage points.**	
							**Knowledge:** timing of the fertile window (proportion answering single question correctly)	The intervention group had a significantly higher proportion of students answering correctly than the control (72% vs 32%), a difference of **40 percentage points**.	
Nguyen 2015	Vietnam	Not reported	Non-randomized trial	**Total**: 928 (Intervention: 459, Control: 469) Adolescent school students. Urban and rural settings.	**Intervention:** SRH education **Control:** No intervention	2 weeks post-intervention	**SRH Knowledge** about the signs of puberty (proportion of students correctly identifying more than 5 signs)	**School 1–- Urban:** The increase in the proportion of students answering correctly was **28.38 percentage points greater** in the intervention group. **School 2–- Rural:** The increase in the proportion of students answering correctly was **13.43 percentage points greater** in the intervention group.	
							**SRH Knowledge** Knowledge that pregnancy is more likely some days of the menstrual cycle (proportion answering single question correctly)	**School 1- Urban:** The increase in the proportion of students answering correctly was **42.16 percentage points greater** in the intervention group. **School 2 – rural:** The increase in the proportion of students answering correctly was **37.91 percentage points greater** in the intervention group.	
Greaves 2019	Vanuatu	2019	Pre-post	**Total:** 82 (25 follow-up) Female athletes. Age 13-59 ($\bar{x}$=22) (89% 13-29).	**Intervention:** Menstrual product trial + MH education **No control**	After the 4-month trial period	**Missing sport due to period**	Of 82 participants at baseline, 31% reported never missing sport due to menstruation. Of 25 participants completing the endline survey, 72% reported not missing sport in the 4 months post-intervention. No further analyses/data provided.	

Materials, facilities, and services

Study ID	Country	Timeframe	Study type	Participants	Intervention and control	Time to follow up	Outcomes	Study Findings	Risk of Bias
Downing 2021	Vanuatu	2019	RCT	**Total:** 192 (136 follow up). Women and girls in disaster effected settings. Age 15-45. Rural and urban settings	**Intervention type:** Comparison of: 2 brands of disposable pad, 2 brands of reusable pads.	2 months post-intervention	**Acceptability of sanitary product**	All four products indicated good level of acceptability (range 80-97%)	
Weerawatsopon 2020	Thailand	2019-2020	Randomized Crossover Trial	**Total**: 98 (Group A: 49, Group B: 49) Female health care personnel. Age 18 – 50 ($\bar{x}$: 32). Urban Hospital setting.	**Intervention:** 6-month menstrual cups trial. Instruction manual and instructional video provided. Group A trialled sanitary pads cycles 1st – 3rd and menstrual cups 4th – 6th cycles. Group B in reverse.	At 3 months and 6 months (post intervention)	**Product satisfaction**	At both 3 and 6 month data collection, participants reported the median score for menstrual cups as 1-point higher than sanitary pads on a 5-point Likert scale.	
							**Acceptability of menstrual cup**	89% Acceptability (no comparison between groups)	
Grant 2020	Loas	2017-2019	Pre-post	10 Schools<br>**Total:** 292 (Midterm: 291, Endline: 286) Adolescent school students. Aged 10-19. Rural setting.	**Intervention type:** WASH infrastructure + training to sew reusable pads (+ education)<br><br>**No control**	Immediate, midline& endline	**School absenteeism due to menstruation:** Self-reported through a single question.	Study authors state: *"This indicator provides no meaningful data. The percentage was already extremely low at baseline."*	
Greaves 2019	Vanuatu	2019	Pre-post	**Total:** 82 (25 follow-up)<br>Female athletes.<br>Age 13-59 ($\bar{x}$=22) (89% 13-29).	**Intervention:** In-person MHM education session. Duration not reported. The participants trialled 2 x pairs of menstrual underwear or 1 x pair and 1x menstrual cup over 4 months.<br><br>**No control**	After the 4-month trial period	**Missing sport due to period**	Of 82 participants at baseline, 31% reported never missing sport due to menstruation. Of 25 participants completing the endline survey, 72% reported not missing sport in the 4 months post-intervention.<br>No further analyses/data provided.	

Care for discomforts and disorders

Study ID	Country	Timeframe	Study type	Participants	Intervention and control	Time to follow up	Outcomes	Study Findings	Risk of Bias
Al Ajeel 2020	Malysia	2017-2018	CBA	**Total:** 480 (Intervention: 268, Control: 212) Female students with Primary Dysmenorrhea Age 13-18.	**Intervention:** Interactive education sessions<br><br>**Control.** No intervention. Booklet provided at end of study.	2 months post-intervention	**PD Knowledge** (quiz scored from 0-20)	Mean knowledge score increased in both intervention and control groups. The intervention group increase was significantly greater, with a 3.02 point increase beyond the control. As a percentage score, this represents a greater **increase of 15 percentage points**.	
							**PD Self-care** (likert-scored items, total score from 40-200)	Self-care scores increased in both groups, with a significantly greater increase in the intervention group, with an 18.21 point increase beyond the control.	
Juan 2016	China	2014-2015	cRCT	**Total:** 391 (Intervention 195, Control: 196) Female university students with PD. Age 18-20.	**Intervention type:** Interactive education session and self-management intervention<br><br>**Control:** No intervention.	Followed up for 6 months (observation period)	**PD Symptoms**	Intervention group PD symptom score mean reduced by 2.32, while the control mean decreased by 0.39, a difference of 1.93 which was statistically significant. Standardised Mean difference (endline) = d=-0.62, 95%CI -0.82,-0.41.	
							**Pain** (likert scale from 0-10)	The decline in mean pain rating over time (3 and 6 month observation periods) for the intervention group was (1.21), and for the control group (0.27), with the 0.94 greater reduction statistically significant.	
							**QoL** (measured by SF-36)	The intervention group improved both physical health 3.63 (SD 1.08) and mental health 2.19 (SD 1.56) scores over the 6-month observation period, and were statistically significant.<br>The control group reported significantly improved physical health by a mean 0.74 (SD 0.37), but not mental health (0.46, SD 0.27).	
Su 2016	China	Not reported	CBA	**Total:** 128 (116 final sample) (Intervention: 66, Control: 62). Post-menarchal adolescent girls from two schools. Aged 12-15. Urban setting.	**Intervention-type:** Interactive education sessions<br><br>**Control:** No intervention	Immediate	**Confidence for Menstrual Health Care**	The intervention group experienced a mean 0.23 greater increase in confidence score than the control. Authors report this is statistically significant, however calculation of standardised mean difference finds d=0.22 (95%CI -0.13, 0.56)	
							**Pain Self-Care behaviour**	The intervention group reported improved self-care behaviour scores, with a 0.24 greater increase than the control group. Authors report this difference is statistically significant, however calculation of standardised mean difference finds d=0.16 (95%CI -0.19, 0.50).	
Djupri 2022	Indonesia	Not reported	CBA	**Total:** 58 (Intervention: 29, Control: 29) Female students. Age 16-17. Urban Vocational School.	**Intervention type:** Pelvic rocking<br><br>**Control:** Not reported. Assumed no intervention.	Immediate	**Pain** (rated on scale from 1-10)	The intervention group experienced a decrease in 2.4 pain score, while the control experienced only a 0.9 decrease. A **total 1.5 point improvement** for the intervention beyond the control.	
Bustan 2018	Indonesia	Not reported	CBA	**Total:** 96 (Intervention: 48, Control: 48) Rural female nursing students with PD in past 6-months. Age 18-19.	**Intervention:** Abdominal stretching<br><br>**Control:** No intervention	After the 3-week intervention period	**Pain** (rated on scale from 0-10)	The intervention group reported reduced pain scores by 1.85, while control group reduced by 0.16. A **total 1.69 point improvement** for the intervention beyond the control.<br>Only means reported. No reported standard deviations.	
Rejeki 2021	Indonesia	Not reported	RCT	**Total:** 130 (Intervention: 65, Control: 65) Female adolescent students. Aged 15-17. Urban and rural settings.	**Intervention type:** Abdominal stretching<br><br>**Control:** No intervention	Not reported	**Pain** (rated on numeric scale. Authors do not report scale range/)	The intervention reduced pain scores by 3.12, while control group reduced by 1.01. A **total 2.11 point improvement** for the intervention beyond the control.<br>Only means reported. No reported standard deviations.	

Supportive social environment

Study ID	Country	Timeframe	Study type	Participants	Intervention and control	Time to follow up	Outcomes	Study Findings	Risk of Bias
Grant 2020	Laos	2017-2019	Pre-post	10 Schools<br>**Total:** 292 (Midterm: 291, Endline: 286)<br>Adolescent school students. Aged 10-19. Rural setting.	**Intervention:** Teacher and Youth leader training on MH Education Session (+ broader WASH)<br><br>**No control**	Immediately after workshop.<br><br>Midline.<br><br>Endline conducted end of 2019	**MHH Knowledge and Attitudes (combined as a single outcome)**	*"Pre- and post-test comparison showed 94% of participants improved knowledge and attitudes of MHM with an average improvement rate of 56% on a 6-point scale. The average rate of improvement was 2.6 points, or almost 50%."*<br>No further data provided.	
							**Seeking social support**	The proportion of participants who reported talking to a teacher before their first period increased from 1% to 49% although the time to menarche in the sample was unclear. The proportion of respondents reporting they would talk to someone about menstruation before their first period increased from 36% to 49% and the proportion reporting they would talk to a teacher increased from 0% to 57%.	
							**School absenteeism due to menstruation**	Study authors state: *"This indicator provides no meaningful data. The percentage was already extremely low at baseline."*	
UnTold Research (UNICEF) 2021	Indonesia	2019-2020	CBA	25 schools: (Intervention: 20 Control: 5)<br>Student sample: (Baseline: 911, endline 762).<br>Urban setting.	**Intervention:** MH education + storybook<br><br>**Control:** No control group	Immediate	Evaluation included broader health outcomes;<br><br>MHH-related outcomes included:<br>**Menstrual Knowledge**	The proportion of students with mid to high knowledge increased from 56 to 65% in the intervention group, and 66% to 68% in the control, a difference in change of 7%. *Dropout was substantial and it appears missing data were not imputed. No null-hypothesis significance tests were undertaken.	
							**Menstrual Knowledge and Perceptions**	Review authors were unable to determine whether attitudes (perceptions) were included in the composite knowledge score.	
							**Comfort discussing menstruation**	Girls exposed to intervention programming showed significant (5%) higher agreement (58%, compared to a baseline of 53%) that they have a person they can trust to ask questions about their body, compared to girls attending control schools who showed no significant increase in agreement (51%, compared to a baseline of 53%) (2%)<br>Intervention group increased self-reported comfort discussing menstruation with the following individuals, beyond the change reported in the control group:<br>Sister: 0%<br>Mother: -8%<br>Female friends: 3%<br>Female teachers: 6%<br>Health Officer: 5%<br>No statistical significance tests were undertaken.	

Judgement

Critical

Serious (High)

Moderate (Some Concerns)

Low

No Information
